# Large-scale survey of prion protein genetic variability in scrapie disease-free goats from the United States

**DOI:** 10.1371/journal.pone.0254998

**Published:** 2021-07-19

**Authors:** Mohamed Zeineldin, Kimberly Lehman, Natalie Urie, Matthew Branan, Alyson Wiedenheft, Katherine Marshall, Suelee Robbe-Austerman, Tyler Thacker

**Affiliations:** 1 National Veterinary Services Laboratories, Veterinary Services, Animal and Plant Health Inspection Service, United States Department of Agriculture, Ames, IA, United States of America; 2 Department of Animal Medicine, College of Veterinary Medicine, Benha University, Benha, Egypt; 3 National Animal Health Monitoring System, Veterinary Services, Animal and Plant Health Inspection Service, United States Department of Agriculture, Fort Collins, CO, United States of America; 4 Department of Clinical Sciences, Colorado State University, Fort Collins, CO, United States of America; Deutsches Zentrum fur Neurodegenerative Erkrankungen, GERMANY

## Abstract

Scrapie is a slowly progressive neurodegenerative disease of small ruminants caused by an accumulation of an abnormal isoform of prion protein in the central nervous system. Polymorphisms of the prion protein gene (PRNP) strongly modulate scrapie resistance and incubation period in goats. The aim of this study was to identify PRNP genetic variability in goats across the United States. Blood from a total of 6,029 apparent scrapie disease-free goats from 654 operations and 19 breeds were analyzed. Sequencing of PRNP revealed 26 genotypes with different rates based on eight codons. The GG127, RR154, and QQ222 genotypes were predominant and showed a remarkably high rate across all goats. The QK222 and NS146 genotypes, known to be protective against scrapie, were found in 0.6% [with 95% CI = (0.3, 1.2)] and 22.0% [95% CI = (19.1, 25.2)] of goats, respectively. The QK222 genotype was found in 23.1% of Oberhasli goats tested, with 95%CI = (3.9, 68.7)] and 22.0% of Toggenburg goats tested with 95%CI = (9.7, 42.5)], while NS146 was found in 65.5% of Savannah goats tested, with 95%CI = (30.8, 89.9), 36.7% of Boer goats tested, with 95%CI = (33.1, 40.4), 36.3% of Nubian goats tested, with 95%CI = (27.0, 46.7)], and 35.6% of LaMancha goats tested, with 95%CI = (22.8, 50.8%). The MM142 and IM142 genotypes were found more frequently in goats on dairy operations, while the HR143, NS146, and ND146 genotypes were found more frequently in goats on meat operations. Goats in the east region had a higher percentage of goats with RH154, RQ211, and QK222 genotypes than goats in the west region. The results of this study showed high genetic variability of PRNP among the U.S. goat population, with differences by location and breed, and may serve as a rationale for development of goat breeding programs at the national level to mitigate the risk of scrapie.

## Introduction

The goat population in the United States (U.S.) totaled 2.6 million head on over 136,000 operations on January 1, 2020 [1], making it a noteworthy source of milk and meat for human consumption. Goat-producing agricultural systems in the U.S. generally involve a variety of goat breeds in various production systems including meat production, dairy production, angora/cashmere production, brush control, and show or companion goats. Therefore, surveillance of the U.S. goat population is valuable to investigate goat genetics and susceptibility to pertinent diseases, such as scrapie.

Scrapie, a transmissible spongiform encephalopathy (TSE), is a fatal, slowly progressive neurodegenerative disease affecting sheep and goats [2–5]. TSEs affect a variety of mammalian species including humans [e.g. Creutzfeldt–Jakob disease (CJD)]; [6], cattle [e.g. bovine spongiform encephalopathy (BSE)]; [7], and deer and elk [e.g. chronic wasting disease (CWD)]; [8]. Scrapie was first reported in U.S. sheep and goats in 1947 and 1969, respectively, and has since been considered a major health issue in both small ruminants. The presence of scrapie in the U.S. costs around $20 million annually due to significant production losses, export loss of sheep and goat products including genetic materials, and increased expenditures for carcass disposal [9].

Scrapie susceptibility and the incubation period in ruminants has been proven to be largely mediated by polymorphisms in the prion protein gene (PRNP) [3]. A common feature to all TSEs is the accumulation of an abnormal isoform (PrP^Sc^) of the cellular host-encoded prion protein (PrP^C^) in the central nervous system and peripheral tissues [10]. The incubation period of scrapie is typically between 18 months and five years, during which the infected animals can transmit the infectious agent to other animals [11]. Clinical signs of scrapie in sheep and goats include head and neck tremors, changes in gait, progressive weight loss despite retention of appetite, behavioral changes, lip smacking, teeth grinding, increased sensitivity to noise, skin biting, and excessive salivation [12]. The characteristic lesions seen in the central nervous system include widespread neuronal degeneration, spongiform disintegration, and prominent astrocytosis [13].

Scrapie in goats has been reported in Greece [3], France [14,15], United Kingdom [4,16,17], Japan [18,19], Korea [20], Netherlands [21], Cyprus [17,22], Canada [23], Italy [24] and the United States [25]. In goats, previous research has revealed polymorphisms in a number of PRNP codons are associated with susceptibility to disease including 21, 24, 37, 49, 102, 110, 127, 133, 137, 142, 143, 146, 151, 154, 168, 211, 218, 220, 222, and 240 [4,24,26].

To the best of our knowledge, large scale association studies between PRNP genetic variability and polymorphisms that exhibit a role on resistance or susceptibility to scrapie in the U.S. goat population are limited. However, some studies have reported the genetic variability and polymorphism of the PRNP gene in goats. Resistance to natural scrapie development and increased scrapie incubation periods in goats has been associated with PrP variants. The GS127, M142, Q211, and HR154 polymorphic variants are associated with prolonged incubation periods [14]. The S146 and K222 variants have been associated with scrapie resistance after experimental challenge in which none of the NS146 or QK222 goats had tissue accumulation of PrP^Sc^ or developed clinical signs of scrapie for 7.5 years [25]. Further research on the PRNP codon 222 indicated all goats that were diagnosed with classical scrapie were almost entirely composed of the QQ222 variant [27].

While there is evidence that goat genetics play an important role in scrapie susceptibility, no related management measures have been implemented. To determine the viability of breeding goats for scrapie resistance, a large-scale goat surveillance program to determine the overall scrapie susceptibility in the U.S. goat population was needed. The aim of this study was to identify PRNP genetic variability in goat breeds raised in the U.S. to assess its likely impact on scrapie occurrence in goats and evaluate the potential of implementing breeding management programs to enhance the prevalence of scrapie resistant variants. This study was part of the National Animal Health Monitoring System (NAHMS) Goat 2019 study, which was a two-phase cross-sectional study to provide essential information on goat health and management.

## Materials and methods

### Study design

NAHMS is a nonregulatory program within the U.S. Department of Agriculture, Animal and Plant Health Inspection Service, Veterinary Services [28], that was initiated in 1983 to collect, analyze, and disseminate data on animal health, management, and productivity across the United States. NAHMS has been conducting national studies to collect detailed health and production information for livestock, poultry, equine, and aquaculture periodically, and on a rotating basis, since 1990. Producer participation in all NAHMS studies is voluntary; and, to protect producer identities, individual operation data are kept confidential. In 2019, NAHMS conducted a national cross-sectional study to collect health and production information about the U.S. goat industry. A subset of operations volunteered to allow for biologic sample collection, including blood, from their goats. Generally, NAHMS analyses of study data take into account the complex survey sampling design by accounting for stratification, sampling without replacement, and unequal probabilities of selection and nonresponse using adjusted sampling weights so that inference reflects the population from which the sample was initially drawn [see [29] for an overview of statistical survey sampling methodology and [28] for the specific methodology used for the 2019 NAHMS Goat study].

### Data collection and biological sampling

**Fig 1:**
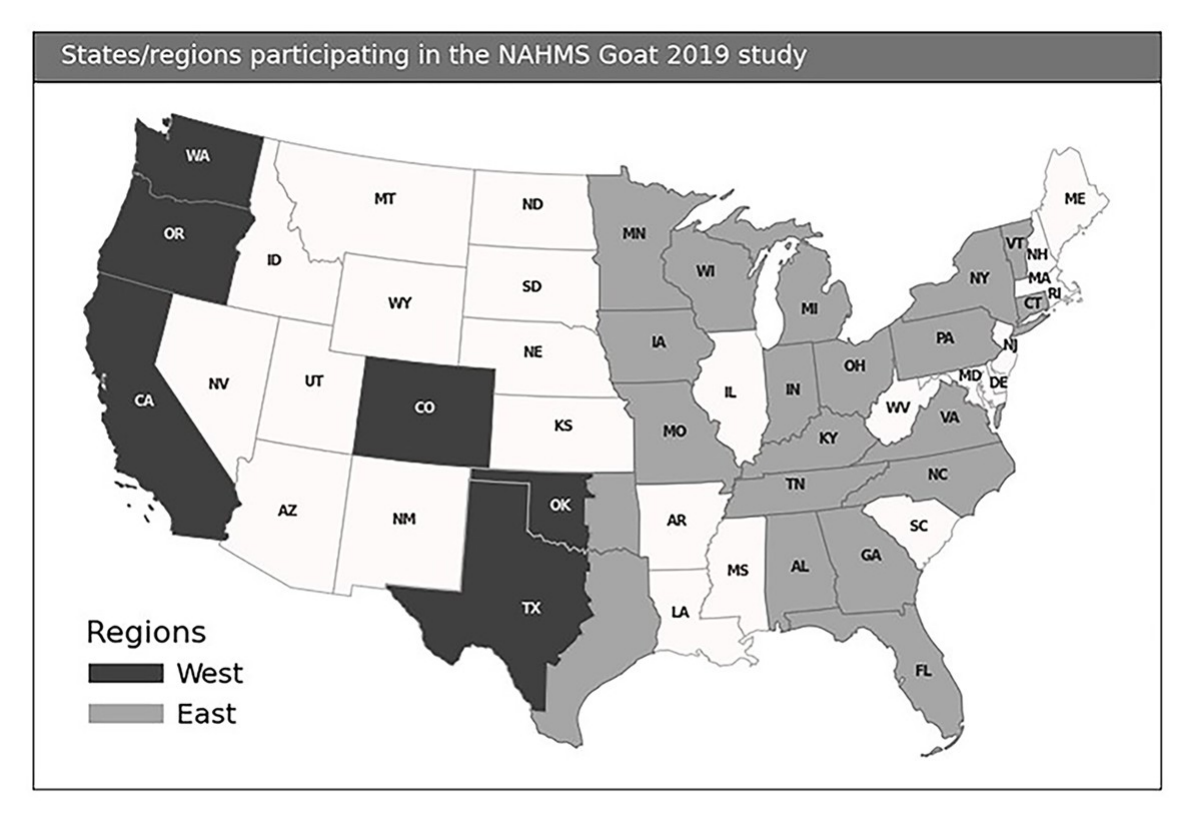
Map of the states and regions for the NAHMS Goat 2019 study. To reflect climate variation and production management, Texas and Oklahoma were divided between the east and west regions roughly on a line corresponding to north-south Interstate 35. The western halves of the states were included in the west region, and the eastern halves were included in the east region. The used and displayed data in that map is the 20m US County boundaries shapefile and are available here: Cartographic Boundary Shapefiles (census.gov).

Data were collected on U.S. goat operations participating in the NAHMS Goat 2019 study. The national adult and kid goat inventory in January 2019 was 2.6 million head on approximately 136,000 operations [1]. The NAHMS Goat 2019 study included 24 states, representing 76.6 percent of U.S. goat operations with five or more adult goats and 82.3 percent of goats on these operations were tested. This study targeted operations with five or more adult goats to reduce the percentage of operations included in the study that were non-commercial (e.g., kept goats as pets). Participating states were categorized into two regions: west (California, Colorado, Oklahoma^1^, Oregon, Texas^1^, and Washington) and east (Alabama, Connecticut, Florida, Georgia, Indiana, Iowa, Kentucky, Michigan, Minnesota, Missouri, New York, North Carolina, Ohio, Oklahoma, Pennsylvania, Tennessee, Texas, Vermont, Virginia, Wisconsin; Fig 1).

For the study, operations selected from the National Agricultural Statistics Service (NASS) list frame were stratified by state, size, and primary production of the operation (meat, dairy, and other). To ensure larger operations and dairy operations were included in the study, they were selected with a higher probability than would have occurred with simple random sample selection. Producers with 5 or more goats were personally interviewed by NASS enumerators from July 1st through August 9th, 2019. All producers completing the NAHMS Goat Management Questionnaire were then invited to complete the Veterinary Services (VS) Questionnaire and biologic sampling. The second interview was conducted by federal or State veterinary medical officers or animal health technicians from September 10th, 2019, through March 20th, 2020. Each operation was assigned a herd size based on their July 1, 2019, inventory of goats and kids reported in the Goat Management Questionnaire. Operations were placed into three size categories: small (5–19 head), medium (20–99 head), and large (100 or more head). Additionally, each operation was assigned a primary production type based on the most common commercial reason that the operation kept goats as reported in the Goat Management Questionnaire. Primary production types were meat, dairy, and other (including angora/fiber). All questionnaires, collection records, study reports, and study methodologies for the NAHMS Goat 2019 Study can be reviewed online (https://www.aphis.usda.gov/aphis/ourfocus/animalhealth/monitoring-and-surveillance/nahms/nahms_goat_studies). Operations that completed the VS Questionnaire were offered biologic testing, which included blood collected from up to 15 goats that were at least 15 months of age. Samples were collected from no more than 5 unrelated bucks and 5 unrelated does (regardless of gestational status) of one breed on each operation. If an operation had more than one breed present, they submitted an additional 5 samples from unrelated bucks or does from the other breed(s). Goats were considered unrelated if they did not share the same sire. Blood samples, up to 7 mL per goat, were collected by USDA veterinary medical officers or animal health technicians using 20-gauge needles and EDTA whole blood vacutainer tubes. Samples were labeled and shipped with ice packs to the USDA National Veterinary Services Laboratories (NVSL) in Ames, Iowa, USA, where processing and testing occurred.

### Biological testing: Genomic DNA extraction, PCR and sequencing

DNA was extracted from all blood samples using the MagMax Core bead extraction kit (Thermo Fisher Scientific, Waltham, MA) following the manufacturer’s protocols. Briefly, blood samples were bead beaten in 0.1mm glass beads for 2 minutes in the Biospec 48 tube bead beater and centrifuged for 10 minutes at 13000 x rpm. Following centrifugation, 200 μl of sample from those tubes were transferred to a deep well plate with 30 μl of bead/Proteinase K mixture and 700 ul of the Lysis-binding solution. Finally, the deep well plate was loaded on automated MagMax 96 machine for binding, washing, and elution of the DNA using MagMax CORE Flex program (Thermo Fisher Scientific, Waltham, MA). The concentration of DNA was assessed using a Qubit (Life Technologies, Grand Island, NY, US) with the High Sensitivity DNA Kit (Agilent Technologies, Santa Clara, CA, US) and stored at -20°C pending analysis. Approximately 1 μg genomic DNA was used as a template for PCR amplification of the open reading frame of PRNP using the primers PrP uniF (5′-AGTCAGTGGAACAAGCCCAG-3′) and PrP_uniR (5′-TGAGGAGGATCACAGGAGGG-3′). The PCR product included PRNP codons (127, 142, 143, 146, 154, 211, 222 and 240). PCR conditions were 95°C for 5 minutes followed by 35 cycles of 94°C for 30 seconds, 56°C for 30 seconds, and 72°C for 30 seconds. This was followed by an extension incubation at 72°C for 10 minutes. Amplified DNA was purified and cleaned before running the sequencing reaction using ExoSAP-IT (Thermo Scientific™, US). PCR fragments were then sequenced using an Applied Biosystems ABI 3500xL genetic analyzer (Applied Biosystems, Foster City, CA, US) using dye-terminating sequence reaction. Briefly, the sequencing reaction was made using the ABI BigDye Terminator v3.1 cycle sequencing kit, following manufacturer instructions. Reactions were cleaned following the BigDye XTerminator Purification Kit product insert at the 10 μl reaction size, and DNA sequencing was performed on both strands of the purified PCR product.

### Bioinformatic and statistical analysis

#### Sequencing analysis

The sequence data were analyzed for PRNP polymorphisms using Geneious prime software (http://www.geneious.com). DNA sequences were compared and aligned with the *Capra hircus* PrP gene reference sequence (GenBank: HM038415.1). The full open reading frame was examined for amino acid polymorphisms to estimate the genetic variability of PRNP gene in goats based on codons 127, 142, 143, 146, 154, 211, 222 and 240. All sequence data obtained in the current study were uploaded to the sequence read archive on the NCBI website with a bio-project accession number PRJNA728650.

#### Survey-based descriptive analysis

Weighted genotypic and allelic proportions were calculated across all operations, goat breeds (Alpine, Angora, Boer, Cashmere, Fainting goats, Kiko, LaMancha, Nigerian dwarf, Nubian, Oberhasli, Pygmy, Pygora, Saanen, Sable, Savannah, Spanish, Toggenburg, Crossbred, and Other breeds), region of the operation (west and east), primary production of the operation (meat, dairy, and other), and goat sex (does and bucks). Weighted descriptive estimation was carried out using SAS-callable SUDAAN software (version 11.0.1, Research Triangle Institute, 2012; version 9.4, SAS Institute, 2012), which allows for the proper analysis of data from complex surveys by accounting for the study design. The estimation of genotypic and allelic frequencies accounted for stratification by State, operation size, and primary production type of the operation, sampling without replacement, finite population corrections, and unequal probabilities of selection adjusted for nonresponse within each stratum. Standard errors and 95% confidence intervals were computed using Taylor series linearization to reflect measurements of uncertainty in the estimates presented. These adjustments were made so that inference could be generalized to the population of doe and buck goats aged 15 months or older in the states included in the study.

#### Univariate analysis

While descriptive estimates were made using both genotypic and polymorphic variants proportions, statistical comparisons of variants proportions were made between levels of the breakout variables (breed, region, primary production, and gender) because of low proportions of goats being homozygous in the minor variants at each codon. Overall differences among levels of the breakout variables were assessed using log-linear test p-values adjusting for the survey design and weights in SUDAAN [30,31]. For overall tests that were statistically significant at the 0.05 significance level, pairwise comparisons between levels of the given breakout variable with respect to allelic proportions were made using Tukey-Kramer multiple comparisons adjusted p-values from logistic regression models regressing allele presence (0 if the animal had the most common genotype at the given codon and 1 if the animal had a minor allele at the codon) on the significant breakout variable fit using SAS’ SURVEYLOGISTIC procedure, which accounts for the survey design. Statistically significant pairwise comparisons were made at the 0.05 level on the adjusted p-value scale. A capital letter coding is used to indicate whether levels of breakout variables are significantly different from one another using the Tukey-Kramer-adjusted p-values. Levels of the same variable that share a letter are not significantly different, and levels that do not share a letter are significantly different at the 0.05 family-wise significance level. In the results and discussion sections, percentages of goats by breed are reported without making explicit statistical comparisons to other breeds with respect to genotypic or allelic proportions.

#### Intraclass correlation coefficient

In order to assess the clustering of prolonged incubation and resistant genotypes on operations, the intraclass correlation coefficients (ICCs) as described by [32] was performed. The ICC estimate and the variance components estimates are asymptotically equivalent and were identical to the hundredths decimal place in all except for one codon, and so only the Fleiss and Cusick estimates are presented [32]. The ICC estimate was computed as:

$$\rho_{FC}=1-\frac{1}{m(\bar{n}-1)\hat{\pi }(1-\hat{\pi })}\sum_{i=1}^{m}\frac{X_{i}(n_{i}-X_{i})}{n_{i}},$$

Where

- *i* = 1,…,*m* indexes operations and *m* is the number of operations,
- *n_i_* is the operation sample size and $\bar{n}=\frac{1}{m}\sum_{i=1}^{m}n_{i}$ is the average operation sample size,
- *X_i_* is the number of animals on operation *i* with the genotype of interest (e.g., S127),
- $\hat{\pi }$ is the estimate of the prevalence of the genotype of interest among all animals.

The closer *ρ_FC_* is to 0, the lower the correlation among animals on a given operation with respect to presence of the genotype of interest (meaning if one animal has the genotype of interest, the less likely we are to find another animal on the same operation with the genotype of interest). Conversely, the closer *ρ_FC_* is to 1, the higher the correlation among animals on a given operation (meaning if one animal has the genotype of interest on an operation, the more likely we are to find more).

#### Multivariate analysis

To assess the multivariate variability of allelic representations, an Analysis of Molecular Variance (AMOVA) framework was adopted [33]. Using this method, the variance among dissimilarities in individual animal’s allelic representations in multivariate space can be partitioned and attributed to factors of interest. In this case, the stratification variables (State, operation size, and primary production), gender, breed, and operation identifier were used to explore variability between animal-level [34] dissimilarities in multivariate space. The adonis function from the vegan package in R (version 3.5.3; R Core Team, 2019), implemented within R Studio (version 1.1.463; R Studio Team, 2020) was used to fit AMOVA models using the method proposed in [35], which is akin to Multivariate Analysis of Variance (MANOVA) on dissimilarity matrices, though test statistics are estimated using non-parametric permutation tests rather than distributional assumptions. Type I AMOVA sums of squares were used to partition variance and test for the significance of terms, where the order of terms added to the model was the fully crossed stratification variables, gender, breed, State-breed interaction, and operation identifier. Permutations were performed assuming animals within the same sampling stratum were exchangeable. In addition, a multivariate extension of Levene’s test for homogeneity [36] was used to test for the multivariate homogeneity of dispersion among groups defined by goat breed using the betadisper function from the vegan package in R.

To explore the multivariate genetic distance between breeds, correspondence analysis [37] was used to reduce the dimensionality of the weight-scaled allelic rate measures at the breed level and to visualize multivariate distance relationships between breeds and between codons. The correspondence analysis was carried out using the dudi.coa function from the ade4 package [38] in R on the weight-scaled, pairwise Nei distance matrix. To further explore relationships between breeds, a neighbor-joining tree was estimated using the nj function from the ape package in R, which estimates neighbor-joining tree structures [34].

#### Statistical significance as a tool

Statistical significance is a tool used here to focus the analysis and discussion towards common or likely relationships in the population in a complex, multivariate system of genetic expressions in goats within a hierarchical population structure. Relationships that do or do not meet the significance levels are not implied to be present or absent at a practical scale; they just did or did not meet the requirements to be statistically significant given the methods described above. Point estimates and variance estimates are given for the reader to be able to further investigate relationships beyond the binary decisions made regarding statistical significance.

### Descriptions of PRNP genotypes

In text and figures, genotypes are described by the single letter amino acid code: D, aspartic acid; G, glycine; H, histidine; I, isoleucine; K, lysine; M, methionine; N, asparagine; P, proline; Q, glutamine; R, arginine; and S, serine.

## Results

### Sample profile

Samples were collected from a total of 6,029 apparently scrapie disease-free goats (17.6% bucks and 82.4% does) from 654 goat operations with 5 or more goats across the U. S. The total number of samples collected from each operation ranged from 1 to 20, with a mean of 9.2 samples. Overall, 61.7% of the tested goats (62.2% of operations) were located in the east region, and 38.3% of goats (37.8% of operations) were located in the west region. Of the sampled goats, 21.1% (31.7% of operations) were from small operations, 51.1% (46.9% of operations) were from medium operations, and 27.8% (21.4% of operations) were from large operations. Overall, 34.0% of the tested goats (33.6% of operations) were from primarily meat-producing operations, 45.8% of goats (43.9% of operations) were from primarily dairy-producing operations, and 20.2% of goats (22.5% of operations) were from operations with a primary production of other. The goats sampled accounted for 19 different breeds that are commonly reared in the U.S. including: Crossbred (17.8% of tested goats), Boer (16.8%), Nubian (11.9%), Alpine (11.5%), Nigerian dwarf (10.6%), Saanen (7.7%), LaMancha (6.1%), Kiko (2.8%), Toggenburg (2.2%), Spanish (2.2%), Oberhasli (1.8%), Angora (1.5%), Fainting goats (1.5%), Savannah (1.1%), Pygmy (0.9%), Cashmere (0.6%), Sable (0.4%), and Pygora (0.4%), and other breeds not listed (2.3%). The total number of goat breeds collected from each operation ranged from 1 to 8, with a mean of 2.1 breeds per operation site. The average age of the sampled goats was 4.0 years (standard deviation 2.4 years).

**Fig 2:**
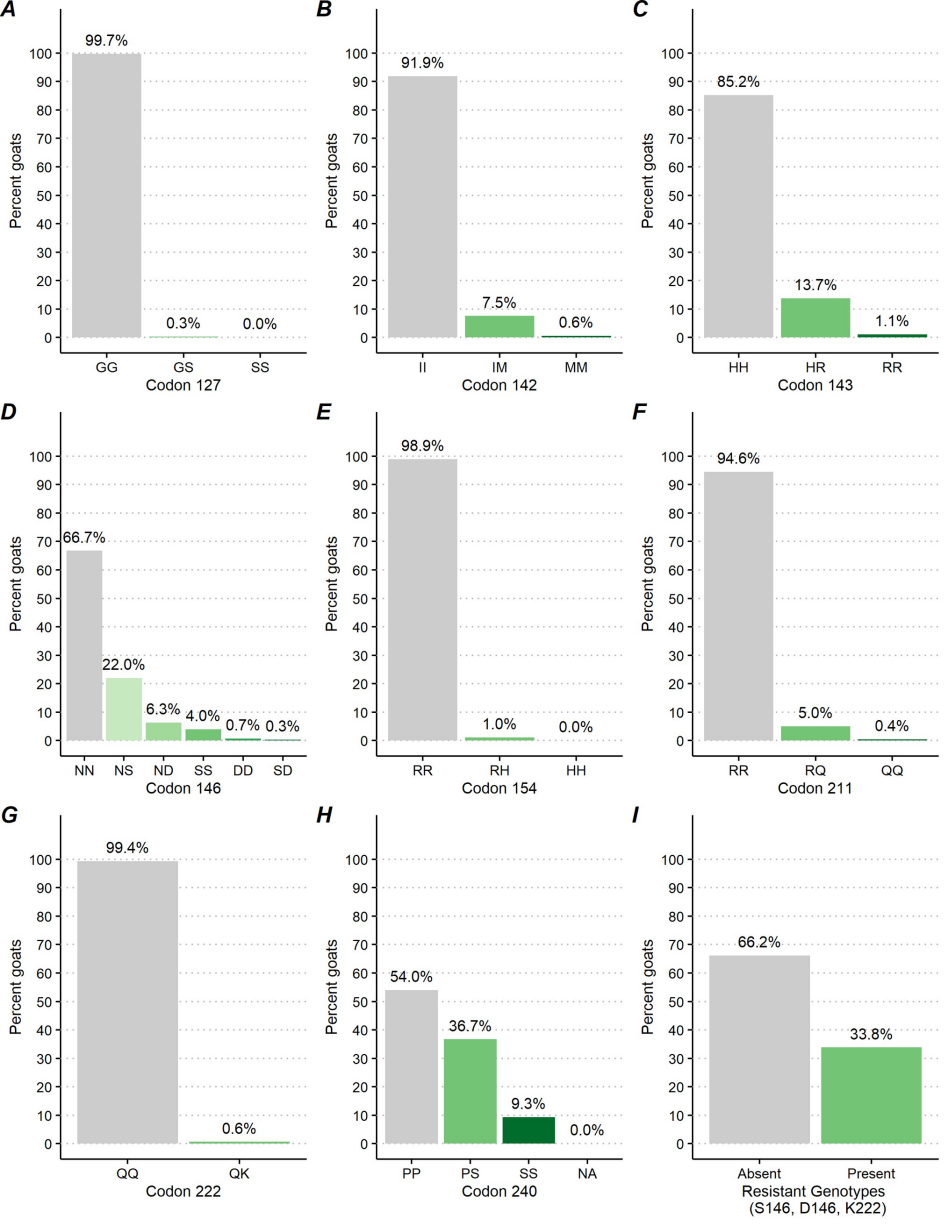
Percentage of goats by caprine PRNP polymorphic variants.

Twenty-six PRNP polymorphic variants at codons 127, 142, 143, 146, 154, 211, 222 and 240 were detected in this study compared to the PRNP reference sequence. The GG127, NN146, RR154 and QQ222 were the predominant variants of the caprine PRNP gene and showed high rate across all goats from the U.S. (Fig 2).

#### Weighted descriptive analysis of genotypic and allelic proportions

**Fig 3:**
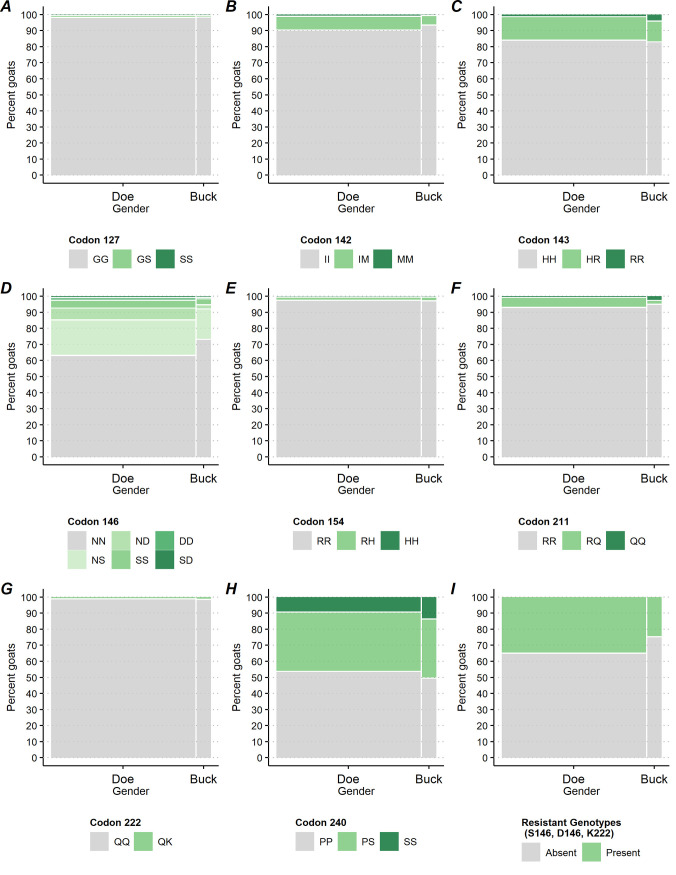
Percentage of goats by caprine PRNP polymorphic variants at codons 127, 142, 143, 146, 154, 211, 222 and 240 and by gender.

**Fig 4:**
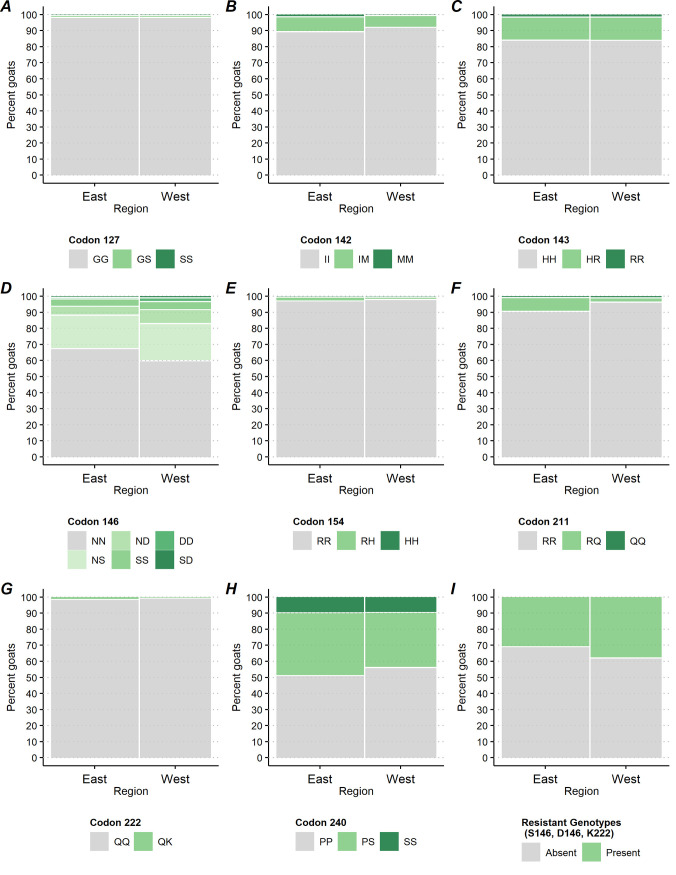
Percentage of goats by caprine PRNP polymorphic variants at codons 127, 142, 143, 146, 154, 211, 222 and 240 and by region.

**Fig 5:**
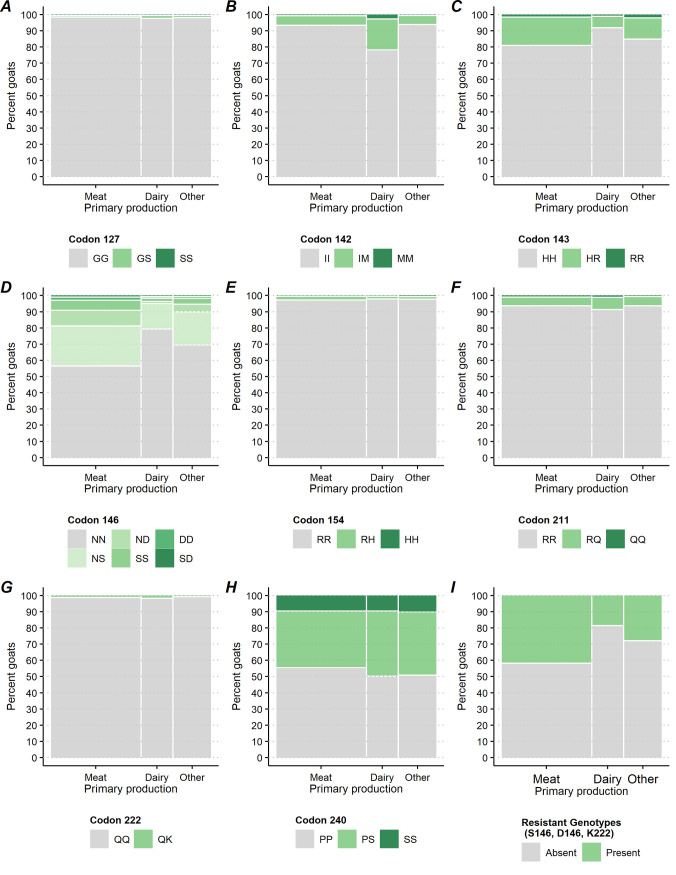
Percentage of goats by caprine PRNP polymorphic variants at codons 127, 142, 143, 146, 154, 211, 222 and 240 and by primary production types.

**Fig 6:**
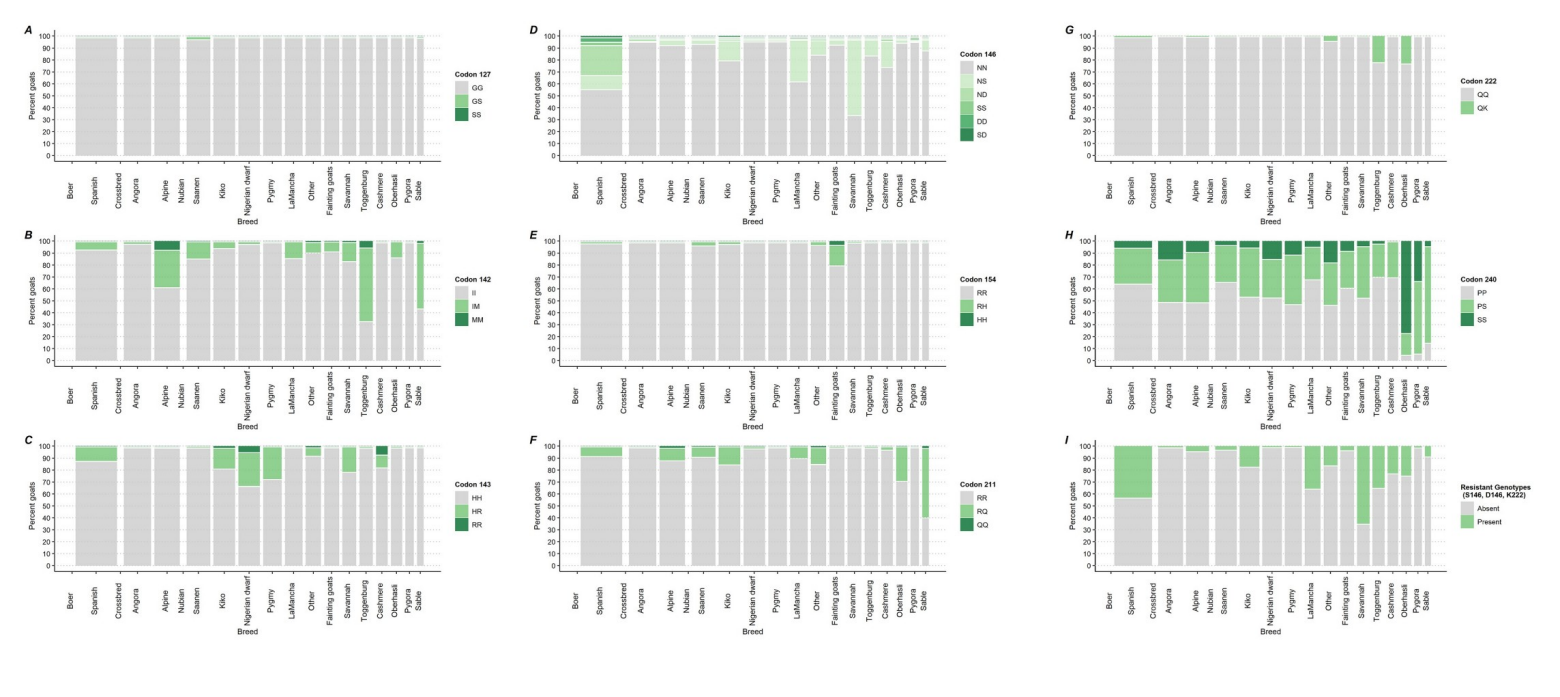
Percentage of goats by caprine PRNP polymorphic variants at codons 127, 142, 143, 146, 154, 211, 222 and 240 by goat breed (widths of bars are taken to be the cubed roots of the weighted frequencies of goats by breed).

**Fig 7:**
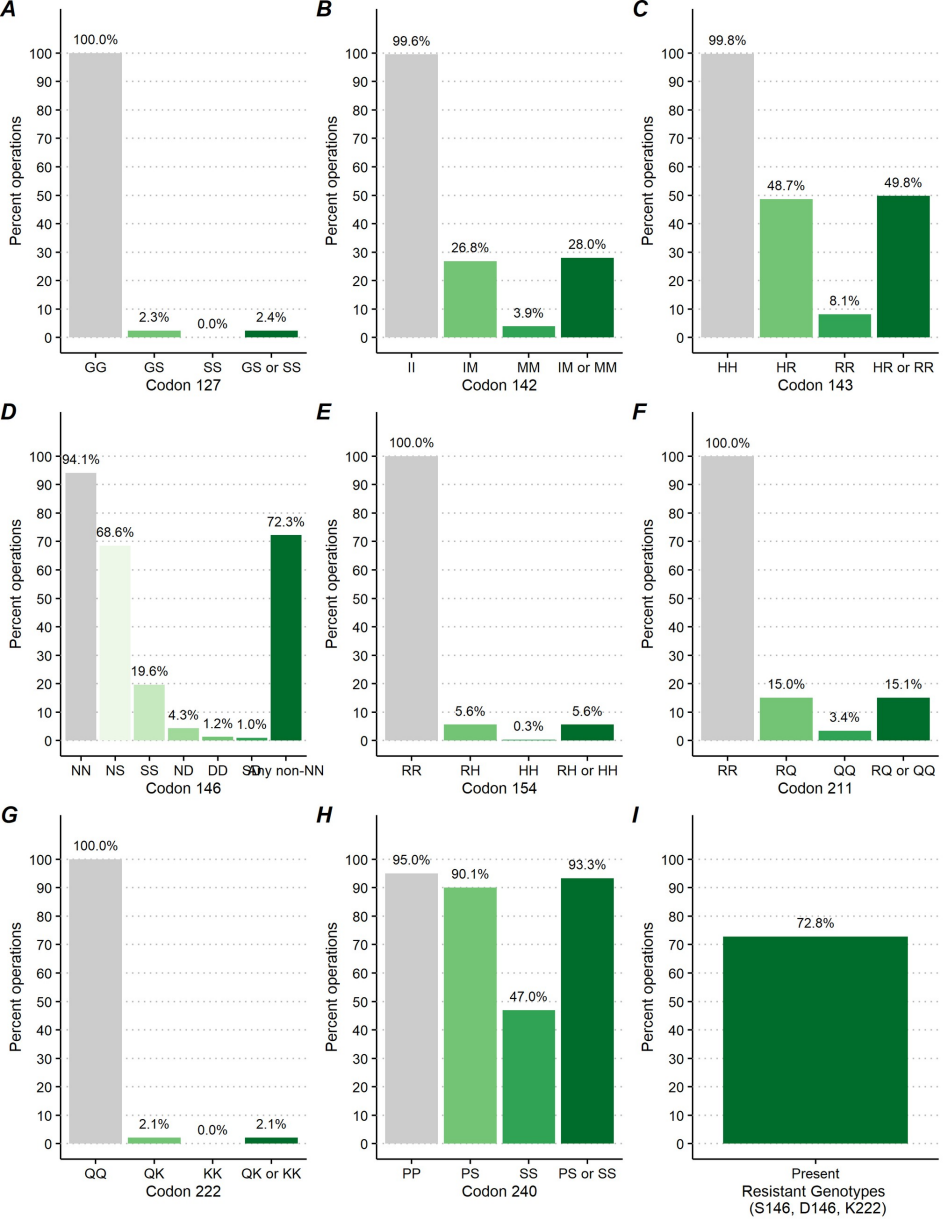
Percentage of goat operations by caprine PRNP polymorphic variants at codons 127, 142, 143, 146, 154, 211, 222 and 240.

**Fig 8:**
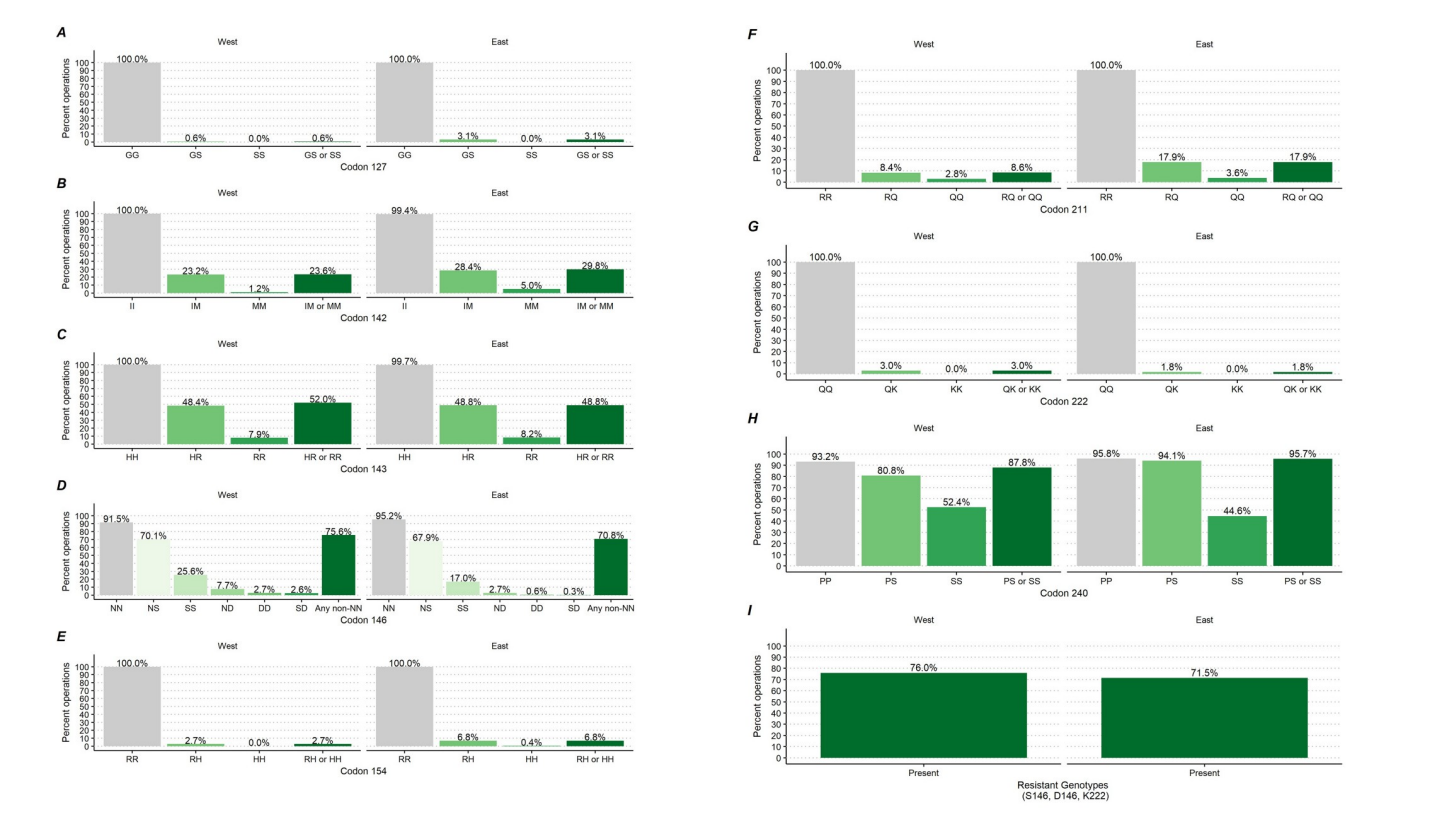
Percentage of goat operations by caprine PRNP polymorphic variants at codons 127, 142, 143, 146, 154, 211, 222 and 240 and by region.

**Fig 9:**
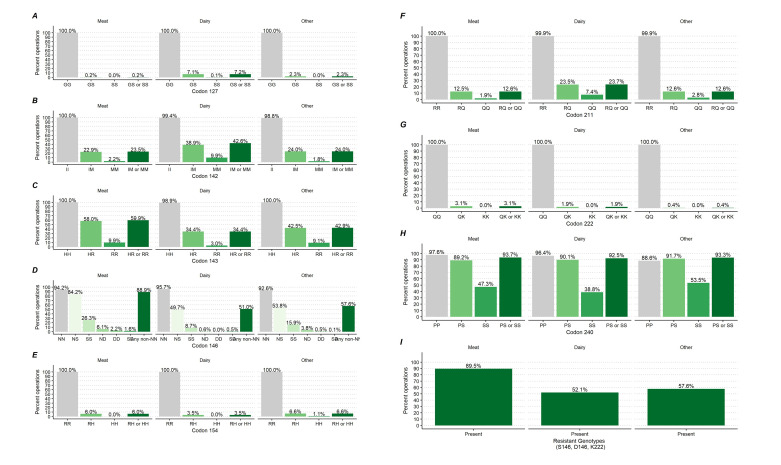
Percentage of goat operations by caprine PRNP polymorphic variants at codons 127, 142, 143, 146, 154, 211, 222 and 240 and by primary production.

Descriptive estimates of percentages of goats and goat operations with specific genotypes, by breakout variable and by codon follow, along with the results from tests of significance of the effects of each of the breakout variables (gender, region, primary production, and breed) on the presence of the prolonged incubation or resistant genotypes at the animal and operation levels, by codon (Tables 1 and 2). Full sets of descriptive statistics for percentages of goats by genotype, breakout variable, and codon can be found in Figs 3–6 and in S1A–S1I Table and similar statistics for percentages of operations can be found in Figs 7–9 and in S2A–S2I Table.

**Table 1:** Proportion of goats with genotypes associated with prolonged incubation period or resistant genotypes, by codon along with significance test results for overall effect of each breakout variable (gender, region, primary production, and breed).

Codon	127		142		143		146		154		211		222		240		146, 222	
Type of protection	Prolonged Incubation		Prolonged Incubation		Prolonged Incubation		Resistance		Prolonged Incubation		Prolonged Incubation		Resistance		Prolonged Incubation		Resistance	
**Allele**	S		M		R		S, D		H		Q		K		S		S, D, K	
**Overall proportion**	0.3		8.1		14.8		33.3		1.1		5.4		0.6		46.0		33.8	
**Gender (*P* value)**	0.013		0.081		0.708		0.028		0.600		0.398		0.442		0.392		0.033	
**Doe**	0.3	a	8.4		14.7		34.2	a	1.0		5.6		0.6		45.7		34.7	a
**Buck**	0.1	ab	5.1		15.8		23.6	ab	1.3		3.6		1.0		49.9		24.4	ab
**Region (*P* value)**	0.881		0.232		0.974		0.077		0.034		0.002		0.005		0.135		0.113	
**West**	0.3		6.6		14.8		37.6		0.5	a	2.2	a	0.1	a	43.2		37.7	
**East**	0.3		9.3		14.8		29.7		1.5	ab	8.1	ab	1.0	ab	48.3		30.7	
**Primary production (*P* value)**	0.015		<0.001		<0.001		<0.001		0.415		0.401		0.133		0.240		<0.001	
**Meat**	0.0	a	5.2	a	17.9	a	41.1	a	1.3		5.0		0.6		43.9		41.7	a
**Dairy**	0.8	ab	20.6	ab	6.7	ab	17.1	ab	0.7		7.3		1.1		49.3		18.2	ab
**Other**	0.4	ab	4.8	ab	13.9	ab	27.5	a	0.9		4.9		0.1		48.6		27.6	ab
**Breed (*P* value)**	0.187		0.003		<0.001		<0.001		0.207		<0.001		0.805		<0.001		<0.001	
**Alpine**	0.0		38.2	abce	0.1	a	3.8	ab	0.1		11.0	abc	0.4		51.2	a	4.2	abc
**Angora**	0.0		1.2	abcd	0.1	a	0.9	a	0.0		0.0	abcd	0.0		50.7	a	0.9	ab
**Boer**	0.1		3.5	abcd	26.6	b	47.0	abc	0.5		2.1	abcdef	0.1		51.4	a	47.0	abcd
**Cashmere**	0.0		0.0	a	17.1	abcd	23.0	abc	0.0		2.0	abcdef	0.0		29.7	a	23.0	abcde
**Fainting goats**	0.0		7.7	abcd	0.0	a	3.4	abcd	19.7		0.4	abcdefg	0.0		38.8	a	3.4	abcdef
**Kiko**	0.0		4.9	abcd	18.0	abcd	17.2	abc	1.3		14.6	abc	0.0		46.3	a	17.2	abcde
**LaMancha**	0.1		13.5	abcd	0.0	a	35.8	abcde	0.0		9.2	abcdef	0.1		31.5	a	35.9	abcde
**Nigerian dwarf**	0.0		1.3	ab	32.9	ab	0.7	a	0.1		0.8	abcde	0.0		47.0	a	0.7	ab
**Nubian**	0.2		10.6	abcd	4.7	abcd	38.6	abcde	0.2		0.3	abcdef	0.0		46.6	a	38.6	abcde
**Oberhasli**	0.0		12.8	abcd	0.4	abc	1.7	ab	0.0		28.5	abc	23.1		95.9	b	24.8	abcde
**Pygmy**	0.0		0.0	a	27.0	abcde	0.6	ab	0.0		0.0	abcd	0.0		52.8	abc	0.6	abc
**Pygora**	0.0		0.0	a	0.0	abcdef	1.1	ab	0.0		0.0	abcd	0.0		94.8	abc	1.1	abc
**Saanen**	1.7		13.8	abcd	0.4	abcd	2.8	ab	2.6		8.1	abcdefg	0.1		33.8	a	2.9	abc
**Sable**	0.5		56.4	abcde	0.0	abcdef	8.5	abc	0.0		59.7	ab	0.0		85.7	ab	8.5	abcde
**Savannah**	0.0		15.9	abcde	20.9	abcd	65.5	abcde	0.4		0.0	abcd	0.1		47.2	abc	65.5	abcde
**Spanish**	0.0		6.0	abcd	11.6	abcde	42.6	abcde	0.8		7.4	abcdef	0.8		35.3	a	43.4	abcdef
**Toggenburg**	0.0		67.2	abcde	0.6	abcd	13.0	abc	0.0		0.5	abcdef	22.0		29.4	a	35.0	abcde
**Crossbred**	0.9		9.7	abcd	12.5	abcde	37.7	abcde	2.0		8.4	abc	0.7		48.8	a	38.3	abcde
**Other**	0.0		8.6	abcd	7.1	abcd	12.3	abbcde	2.3		14.1	abcdef	3.9		53.4	abc	16.2	abcde

Lower case letter coding denotes which levels of a given breakout variable have significantly different proportions of goats with the prolonged incubation or resistant genotypes. Levels that share a letter are not significantly different and levels that do not share a letter are significantly different at the 0.05 significance level with Tukey-Kramer adjustments.

**Table 2:** Proportion of goat operations with any goats with prolonged incubation period or resistant genotypes, by codon along with significance testing for overall effect of each breakout variable (gender, region, primary production, and breed).

Codon	127		142		143		146		154		211		222		240		146, 222	
Type of protection	Prolonged Incubation		Prolonged Incubation		Prolonged Incubation		Resistance		Prolonged Incubation		Prolonged Incubation		Resistance		Prolonged Incubation		Resistance	
**Allele**	S		M		R		S, D		H		Q		K		S		S, D, K	
**Overall proportion**	2.4		28.0		49.8		72.3		5.6		15.1		2.1		93.3		72.8	
**Region (*P* value)**	0.040		0.355		0.681		0.438		0.044		0.018		0.520		0.065		0.463	
**West**	0.6	a	23.6		52.0		75.6		2.7	a	8.6	a	3.0		87.8		76.0	
**East**	3.1	ab	29.8		48.8		70.8		6.8	ab	17.9	ab	1.8		95.7		71.5	
**Primary production (*P* value)**	0.008		0.017		0.014		<0.001		0.572		0.051		0.146		0.964		<0.001	
**Meat**	0.2	a	23.5	a	59.9	a	88.9	a	6.0		12.6		3.1		93.7		89.5	a
**Dairy**	7.2	ab	42.6	ab	34.4	ab	51.0	ab	3.5		23.7		1.9		92.5		52.1	ab
**Other**	2.3	ab	24.0	a	42.9	ab	57.6	ab	6.6		12.6		0.4		93.3		57.6	ab

Capital letter coding denotes which levels of a given breakout variable have significantly different proportions of operations with any goats with the prolonged incubation or resistant genotypes. Levels that share a letter are not significantly different and levels that do not share a letter are significantly different at the 0.05 significance level with Tukey-Kramer adjustments.

#### Codon 127

At codon 127, the G allele was predominant, with 99.7% [95%CI = (99.5, 99.8)] of goats across all operations being GG127 homozygotes. The GS127 heterozygote was detected in only 0.3% [95%CI = (0.2, 0.5)] of goats across 2.3% [95%CI = (1.0, 5.2)] of operations. The SS127 homozygote was extremely rare, occurring in 0.001% [95%CI = (0.0, 0.0)] of goats across 0.0% [95%CI = (0.0, 0.1)] of operations (Figs 2A and 7A).

Testing for differences at the goat level among breakout categories with respect to the GG127 homozygote versus the GS127 and SS127 genotypes, there was no significant difference at codon 127 genotype frequencies between regions (P = 0.881, Fig 4A) and breed (P = 0.187, Fig 6A). There was a significant difference between bucks and does (P = 0.013, Fig 3A) and by type of operation (P = 0.015, Fig 5A). Does presented a higher rate of the GS127 genotype compared to bucks [0.3% with 95%CI = (0.2, 0.6), 0.1% with 95%CI = (0.0, 0.2) respectively]. A higher percentage of goats on dairy operations [0.8% with 95%CI = (0.4, 1.8)] compared to goats on meat operations [0.0% with 95%CI = (0.0, 0.3)] presented with the GS127 genotype (Table 1).

At the goat operation level, a significantly higher percentage of operations in the east region [3.1% with 95%CI = (1.3, 7.3)] had goats with GS127 genotype compared to operations in the west region [0.6% with 95%CI = (0.1, 2.2)] (P = 0.040; Fig 8A). Also, a higher percentage of operations with goats kept primarily for dairy [7.2% with 95%CI = (2.6, 18.8)] had goats with GS127 genotype than operations that kept goats primarily for meat [0.2% with 95%CI = (0.0, 1.6)] (P = 0.008; Fig 9A and Table 2). These differences were driven by the GS127 heterozygote as the SS127 homozygote was very rare (S1A and S2A Table).

#### Codon 142

At codon 142, the majority, 91.9% [95%CI = (89.5, 93.8)], of goats across 99.6% [95%CI = (98.1, 99.9)] of operations were II142 homozygote. The IM142 heterozygote occurred in 7.5% of goats [95%CI = (5.8, 9.7)] across 26.8% [95%CI = (21.1, 33.4)] of operations and the MM142 homozygote occurred in 0.6% [95%CI = (0.3, 1.1)] of goats across 3.9% [95%CI = (2.0, 7.5)] of operations (Figs 2B and 7B).

Comparing IM142 and MM142 to the II142 genotypes at the goat level revealed no significant differences between bucks and does (P = 0.080, Fig 3B), nor goats from east and west regions (P = 0.232, Fig 4B). Goats showed significant differences in genotypic frequencies by operation (P<0.001, Fig 5B) and breed (P = 0.003, Fig 6B). IM142 and MM142 genotypes were found at a higher percentage in goats on dairy operations [20.6% with 95%CI = (15.1, 27.5)] compared to goats on meat operations [5.2% with 95%CI = (3.1, 8.7)]. Higher rates of IM142 and MM142 genotypes occurred in Toggenburg [67.2% with 95%CI = (38.7, 87.0)], Sable [54.9% with 95%CI = (14.2, 90.0)], and Alpine [38.2% with 95%CI = (22.9, 56.3)] goats (S1B Table). Neither of IM142 nor MM142 genotypes were observed in Cashmere, Pygmy or Pygora goats (Table 1).

At the operation level, a higher percentage of dairy operations [42.6% with 95%CI = (31.4, 54.6)] had goats with IM142 or MM142 genotypes compared to operations raising goats primarily for meat [23.5% with 95%CI = (15.9, 33.3)] or other purposes [24.0% with 95%CI = (15.0, 36.1)] (P = 0.017; Fig 8B; S2B Table).

#### Codon 143

At codon 143, 85.2% [95%CI = (82.9, 87.3)] of goats across 99.8% [95%CI = (99.1, 99.9)] of operations were HH143 homozygote. The HR143 heterozygote was detected in 13.7% of goats [95%CI = (11.8, 15.9)] across 48.7% [95%CI = (42.0, 55.4)] of operations. The RR143 homozygote occurred in 1.1% [95%CI = (0.6, 1.8)] of goats across 8.1% [95%CI = (5.3, 12.2)] of operations (Figs 2C and 7C).

At codon 143, There was no significant difference between HR143, RR143, and HH143 genotypes between bucks and does (P = 0.708, Fig 3C) or between goats from the east and west regions (P = 0.974, Fig 4C). The HR143 and RR143 genotypes showed significant differences by operation (P<0.001, Fig 5C) and by breed (P<0.001, Fig 6C). A higher percentage of goats on meat operations [17.9% with 95%CI = (15.0, 21.3)] compared to dairy operations [6.7% with 95%CI = (4.8, 9.2)] were found to have HR143 or RR143 genotypes. Conversely, the HH143 genotype was found at a higher percentage in goats on dairy operations compared to goats on meat operations [93.3% with 95%CI = (90.8, 95.2) versus 82.1% with 95%CI = (78.7, 85.0), respectively, Fig 5C]. The R143 allele was observed in all breeds except Pygora and Sable. HR143 heterozygosity or RR143 homozygosity was represented with higher rate in Nigerian dwarf [32.9% with 95%CI = (25.0, 41.9)], Pygmy [27.0% with 95%CI = (15.7, 42.2)], and Boer goats [26.6% with 95%CI = (22.5, 31.1)] (Fig 6C; Table 1).

At the operation level, 59.9% [95%CI = (48.9, 70.0)] of operations raising goats primarily for meat was significantly higher than the 34.4% [95%CI = (23.1, 47.9)] of dairy operations that had goats with HR143 or RR143 genotypes (P<0.001; Fig 8C; S2C Table).

#### Codon 146

At codon 146, 66.7% [95%CI = (62.2, 71.0)] goats across 94.1% [95%CI = (88.7, 97.0)] of operations were NN146 homozygote. The NS146 heterozygote was observed in 22.0% [95%CI = (19.1, 25.2)] of goats across 68.6% [95%CI = (62.5, 74.1)] of operations and the SS146 homozygote was observed in 4.0% [95%CI = (2.9, 5.5)] of goats across 19.6% [95%CI = (14.5, 26.0)] of operations. The ND146 heterozygote occurred in 6.3% [95%CI = (3.6, 10.7)] of goats across 4.3% [95%CI = (2.4, 7.3)] of operations and the DD homozygote occurred in 0.7% [95%CI = (0.2, 2.3)] of goats on 1.2% [95%CI = (0.4, 3.8)] of operations. The SD heterozygote was detected in and 0.3% [95%CI = (0.1, 1.1)] of goats on 1.0 [95%CI = (0.3, 3.3)] of operations (Figs 2D and 7D).

At codon 146, There was no significant difference in genotype frequencies between regions (P = 0.077, Fig 4D). Genotypes associated with resistance (e.g. NS146, ND146, SS146, DD146, and SD146 genotypes) versus NN146 genotypic rates overall differed significantly by bucks and does (P = 0.028, Fig 3D), type of the operation (P<0.001, Fig 5D), and breed (P<0.001, Fig 6D). A significantly higher percentage of does [34.2% with 95%CI = (29.7, 38.9)] displayed genotypes with resistant alleles compared to bucks [23.6% with 95%CI = (16.7, 32.4)]. The resistant alleles were found less frequently in goats on dairy operations [17.1% with 95%CI = (13.2, 21.9)] compared to goats on meat operations [41.1% with 95%CI = (35.8, 46.7)] or other operations [27.5% with 95%CI = (18.8, 38.2)] (Fig 5D), which was primarily driven by differences in genotypes NS146, ND146, and SS146 (S1D Table). Generally, the resistant genotypes were represented with a greater percentage in Savannah [65.5% with 95%CI = (30.8, 89.0)], Boer [47.0% with 95%CI = (42.9, 51.5)], Spanish [42.6% with 95%CI = (30.1, 56.1)], and Nubian [38.6% with 95%CI = (29.8, 48.2)] goats. Specifically, the biggest driver of this was the resistant NS146 genotype, which was observed in all breeds except Angora and Pygora goats. The NS146 genotype was found at relatively higher frequencies in Savannah [65.5% with 95%CI = (30.8, 89.0)], Boer [36.7% with 95%CI = (33.1, 40.4)], Nubian [36.3% with 95%CI = (27.0, 46.7)] and LaMancha goats [35.6% with 95%CI = (22.8, 50.8)] (S1D Table). The ND146, SD146 DD146, and SS146 genotypes were detected at very low percentages across all the goat breeds with the only notable exceptions being ND146 being detected in 25.4% [95%CI = (15.8, 38.2)] of Spanish goats and SS146 in 8.9% [95%CI = (6.4, 12.5)] of Boer goats (Table 1; S1D Table).

At the operation level, a higher percentage of operations raising goats primarily for meat [88.9% with 95%CI = (80.7, 93.9)] had goats with any resistant genotypes at codon 146 than operations raising goats primarily for dairy [51.0 with 95%CI = (39.1, 62.7)] or other purposes [57.6% with 95%CI = (43.7, 70.4)] (P<0.001; Fig 9D; S2D Table).

#### Codon 154

At codon 154, 98.9% [95%CI = (98.3, 99.3)] of goats across 100.0% of operations showed RR154 homozygote on both alleles at this location of the PrP gene. The RH heterozygote was detected in 1.0% [95%CI = (0.6, 1.6)] of goats across 5.6% [95%CI = (3.3, 9.3)] of operations. The HH homozygote was very rare, occurring in only 0.02% [95%CI = (0.004, 0.204)] of goats across 0.3% [95%CI = (0.0, 2.0)] of operations and was only detected in fainting goat breeds (Figs 2E and 7E).

At codon 154, there was no significant difference in genotype frequencies between bucks and does (P = 0.600, Fig 3E), operation (P = 0.415, Fig 5E), or breed (P = 0.207, Fig 6E). There was a statistically significant difference between the percentage of goats with the RR154 homozygote and those with the RH154 or HH154 genotypes between regions (P = 0.034, Fig 4E), though the difference was small [1.5% with 95%CI = (0.8, 2.6) in the east region versus 0.5% with 95%CI = (0.3, 1.1) in west region]. The predominant genotype at codon 154 across all breeds was RR154 homozygote with 100.0% of Angora, Cashmere, LaMancha, Oberhasli, Pygmy, Pygora, Sable, and Toggenburg goats. The RH154 genotype showed a generally higher percentage in Fainting goats [16.6% with 95%CI = (6.3, 37.1)] (across 46.0% of the 3.1% of operations overall that had any Fainting goats) and less than 3% in all other breeds (Table 1; S1E Table).

At the goat operation level, the 6.8% [95%CI = (3.7, 12.2)] of operations in the east region was significantly higher than the 2.7% [95%CI = (1.3, 5.3)] of operations in the west region with the RH154 or HH154 genotypes (P = 0.044; Fig 8E; S2E Table).

#### Codon 211

At codon 211, 94.6% [95%CI = (92.3, 96.2)] of goats across 100.0% [95%CI = (99.9, 100.0)] of operations were RR211 homozygote. The RQ211 heterozygote occurred in 5.0% [95%CI = (3.5, 7.2)] of goats across 15.0% [95%CI = (11.3, 19.7)] of operations and the QQ211 heterozygote occurred in 0.4% [95%CI = (0.2, 1.1)] of goats across 3.4% [95%CI = (1.4, 7.9)] of operations (Figs 2F and 7F).

At codon 211, There was no significant difference between the RQ211,QQ211 and RR211 genotypic frequencies between bucks and does (P = 0.398, Fig 3F) or primary production types (P = 0.401, Fig 5F). The genotypes RQ211 and QQ211 were displayed at a higher percentage in goats in the east region [8.1% with 95%CI = (5.5, 11.7)] compared with goats in the west region [P = 0.002; Fig 4F; 2.2% with 95%CI = (1.0, 4.5)]. Comparing the genotype frequencies at codon 211 exhibited significant differences among breeds (P<0.001, Fig 6F) with RQ211 and QQ211 genotypes generally observed at higher percentages in Sable [59.7% with 95%CI = (18.0, 90.9)], Oberhasli [28.5% with 95%CI = (9.1, 61.5)], Kiko [5.7% with 95%CI = (6.5, 29.5)], and Alpine [11.0% with 95%CI = (7.0, 16.9)] goats (Table 1; Fig 6F).

At the goat operation level, the percentage of operations in the east region [17.9% with 95%CI = (12.8, 24.4)] with RQ211 or QQ211 genotypes was higher than the same percentage of operations in the west region [8.6% with 95%CI = (5.0, 14.3)] (P = 0.44; Fig 8F; S2F Table).

#### Codon 222

At codon 222, the Q allele predominated across all goats, with 99.4% [95%CI = (98.8, 99.7)] of goats across 100.0% of operations being QQ222 homozygotes. Only 0.6% [95%CI = (0.3, 1.2)] of goats across 2.1% [95%CI = (1.0, 4.6)] of operations were QK222 heterozygotes and no KK222 homozygotes were observed (Figs 2G and 7G).

Comparing the genotype frequencies at codon 222 no significant difference between bucks and does (P = 0.442, Fig 3G), production type (P = 0.0133, Fig 5G), or breeds (P = 0.805, Fig 6G). There was a significant difference in the percentage of the QK222 genotype in goats in the east region [1.0% with 95%CI = (0.5, 2.1)] compared to the west region [0.1% with 95%CI = (0.0, 0.4)] (P = 0.005, Fig 4G; S1G Table).

No significant differences between percentages of operations with QK222 and QQ222 genotypes was observed at the operation level (Table 2; S2G Table).

#### Codon 240

At codon 240, 54.0% [95%CI = (50.6, 57.3)] of goats across 95.0% [95%CI = (90.5, 97.5)] of operations were PP240 homozygous and 36.7% [95%CI = (33.6, 39.8)] of goats across 90.1% [95%CI = (84.5, 93.8)] of operations were PS heterozygous. The SS homozygote was detected in 9.3% [95%CI = (7.9, 11.0)] of goats across 47.0 [95%CI = (40.4, 53.7)] of operations (Figs 2H and 7H).

Comparing the genotype frequencies at codon 240 no significant differences were exhibited across gender (P = 0.392, Fig 3H), region (P = 0.135, Fig 4H), or operation (P = 0.240, Fig 5H). Comparing the genotype frequencies at codon 240 significant differences among breeds was detected (P<0.001, Fig 6H) with generally higher percentages in Oberhasli [78.3% with 95%CI = (60.0, 89.6)] (across 97.9% of the 1.1% of operations overall that had any Oberhasli goats) and Pygora [34.0% with 95%CI = (15.7, 58.7)] goats being SS240 homozygous. SS240 was observed in 10% of goats of Other breeds, Angora, Nigerian dwarf, Nubian, Pygmy, and Boer (Table 1; S1H Table).

No significant differences between percentages of operations with PS240 and SS240 genotypes was observed at the operation level (Table 2; S2H Table).

### Resistant genotypes

Considering the genotypes offering prolonged incubation period, rather than protection, NS146, ND146, SS146, DD146, SD146, and QK222, 33.8% [95%CI = (29.6, 38.3)] of goats across 72.8% [95%CI = (66.9, 78.0)] of operations displayed at least one of the resistant genotypes (Figs 2I and 7I).

Comparing the percentages of goats with any of the resistant genotypes versus the percentages of goats without showed significant differences by gender (P = 0.033, Fig 3I), operation (P<0.001, Fig 5I), and breed (P<0.001, Fig 6I), but no significant differences by region (P = 0.113, Fig 4I). A higher percentage of does [34.7% with 95%CI = (30.3, 39.4)] had a resistant genotype compared to bucks [24.4% with 95%CI = (26.3, 35.4)]. Similarly, a higher percentage of goats on meat operations [41.7% with 95%CI = (36.5, 47.2)] compared to goats on dairy operations [18.2% with 95%CI = (14.2, 23.0)] had any resistant genotype. Savannah [65.5% with 95%CI = (30.8, 89.0)], Boer [47.0% with 95%CI = (42.9, 51.1)], Spanish [34.4 with 95%CI = (30.9, 56.7)], and Nubian [38.6 with 95%CI = (29.8, 48.2)] breeds had higher percentages of goats with any of the resistant genotypes. In addition, over 20% of Crossbred, LaMancha, Toggenburg, Oberhasli, and Cashmere goats had any of the resistant genotypes as well (Table 1; S1I Table).

At the operation level, a higher percentage of meat-producing operations [89.5% with 95%CI = (81.3, 94.4)] had goats with any of the resistant genotypes compared with dairy operations [52.1% with 95%CI = (40.2, 63.7)] or other operations [57.6% with 95%CI = (43.7, 70.4)] (P<0.001; Fig 9I; S2H Table).

#### Resistant genotypes by goat gender

While the majority (99.9%) of operations had does, approximately 63.0% of operations had bucks and 62.8% had both does and bucks. Considering the distribution of resistant genotypes across operations with each type of goat, approximately 69.5% (95%CI = (63.4, 75.0)] of the operations with does had does with any resistant genotype. Approximately 21.3% [95%CI = (16.3, 27.3)] of all operations had resistant bucks. Considering the operations that had both does and bucks, 17.9% [95%CI = (13.3, 23.7)] of operations had both resistant does and resistant bucks. And, considering all operations, 72.8% [95%CI = (66.9, 78.0)] had any goats with any of the resistant genotypes.

### Clustering of genotypes

The ICCs for the prolonged incubation or resistant genotypes at each codon varied from approximately 0.098 for the S127 genotypes to 0.239 for the S146 and N146 genotypes. The ICC for the combined S146, D146, and K222 genotypes was 0.225, and was primarily driven by the genotypes at codon 146 (Table 3).

**Table 3:** Intraclass correlation coefficients (ICC) estimated at the goat operation level, by codon.

Codon	Type of protection	Allele	Intraclass correlation coefficient estimate
**127**	Prolonged incubation	GS, SS	0.098
**142**	Prolonged incubation	IM, MM	0.231
**143**	Prolonged incubation	HR, RR	0.185
**146**	Resistance	NS, ND, SS, DD, SD	0.239
**154**	Prolonged incubation	RH HH	0.146
**211**	Prolonged incubation	RQ, QQ	0.147
**222**	Resistance	QK, KK	0.113
**240**	Prolonged incubation	PS, SS	0.103
**146, 222**	Resistance	NS, ND, SS, DD, SD, QK, KK	0.225

### Population description

Using AMOVA, the percentage of variability in allele presentations were inferred from Nei distances between goats and partitioned by the stratification variables from the sampling design (State in which the animal resided, size of the operation, and primary production of the operation, fully crossed), gender, breed, the State-breed interaction, and operation identifier. Approximately 41.1% of the variability was explained by all the factors taken together. Using Type I AMOVA sums of squares the stratification variables accounted for 11.1% of the variability, gender accounted for less than 0.1%, breed accounted for 10.0%, breed by State accounted for an additional 5.6%, and between-operation variability accounted for the remaining 14.0% of the explained variability. The effects of the stratification, breed, breed-State interaction, and operation were all statistically significant, based on permutation tests (P<0.001). Using Anderson’s multivariate test for homogeneity of dispersion, there were significant differences in dispersion by breed group (P<0.001). Given this observation, in the present study, even differences between breeds with respect to dispersion are nearly as meaningful as differences between breeds with respect to location.

**Fig 10:**
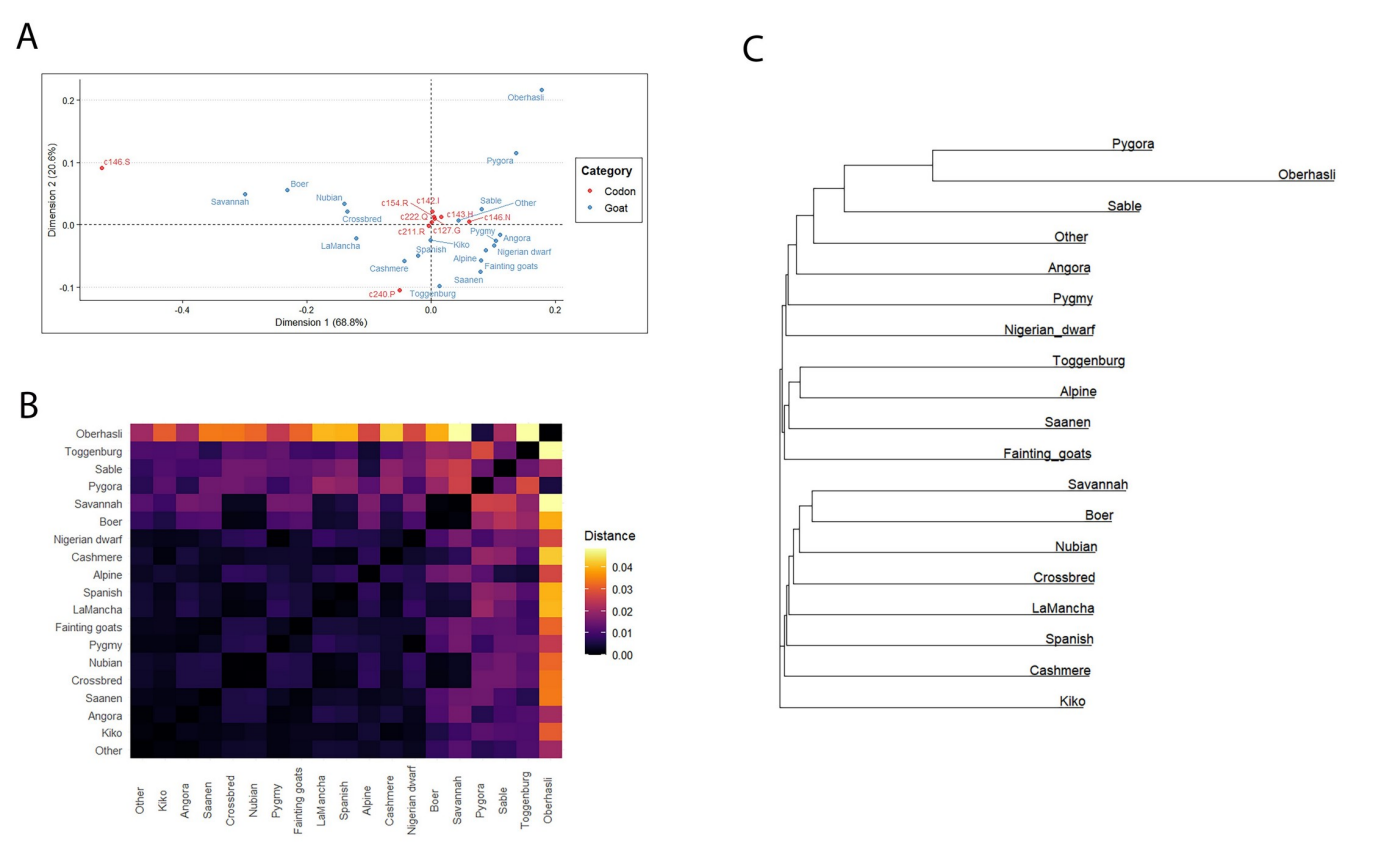
The effect of goat breeds on allelic presentations in the population at the given codon. **(A)** Correspondence analysis plot using the first two dimensions, by category (goat breed and codon). (**B)** Heatmap displaying estimated Nei’s distance between breeds based on allele presence at codons 127, 142, 143, 146, 154, 211, 222, and 240. Rows and columns are sorted by average Nei distance from each other breed. **(C)** Neighbor-joining tree for goat breeds based on estimated Nei’s distance between breeds based on allele presence at codons 127, 142, 143, 146, 154, 211, 222, and 240.

To further develop the analysis of the effect of breed on allelic presentations in the population at the given codons, a correspondence analysis was performed on the breed-level, weighted allelic frequencies. The cumulative projected inertia of the first two dimensions was approximately 89.4% (68.8% and 20.6%), meaning approximately 89.4% of the variability in allelic frequencies was explained by the first two dimensions in the reduction. The goats and the codon alleles were both plotted on the first two dimensions in Fig 10A. The coordinates of the variables (codon alleles) represent the approximate weighting represented in the particular dimension. From Fig 10A, dimensions 1 and 2 are primarily influenced by the differentiation of breeds by their presentation of the S146, the P240, the N146, and the I142 alleles, which is mirrored by the observed variability of descriptive proportions of goats by allele presentations in panels D and H in Fig 6. The coordinates of breeds correspond to the relative distance to other breeds according to the codon allele presentations. For example, Savannah and Boer goats are more similar to one another compared to other breeds primarily because of their similar presentations of goats with S146 alleles. Oberhasli, Pygora, and Sable goats are similar to one another compared to other breeds primarily because of their presentations of goats with S240 alleles. Likewise, Toggenburg goats are different from others primarily on the I142 axis as goats of that breed tended to display a higher rate of M142 alleles compared with goats of other breeds.

Pairwise, weight-scaled Nei distances at the breed level are displayed in Fig 10B in the form of a heatmap and in Fig 10C in the form of a neighbor-joining tree. The relative distance of Oberhosli, Pygora, and Sable breeds are highlighted here, primarily because of the influence of allelic presentations at codon 240. Toggenburg appears distant from a number of other breeds, primarily due to its distance at the M142 codon allele (along with Oberhasli). Similar results can be found in the neighbor-joining tree in Fig 10C, though the bifurcations in the tree diagram can be further explored using the full distance matrix in Fig 10B or the biplot in Fig 10A.

## Discussion

The scrapie eradication program in sheep demonstrated that it is possible to substantially mitigate the prevalence of scrapie using PRNP genotype variabilities [39]; however, resistant alleles were present in over 60% of the US sheep population in the early period of genetic testing for sheep [9]. Despite the low incidence of scrapie in goat population [40], goats may still serve as a scrapie reservoir; therefore, determining the appropriate candidate genotypes present in the goat population will allow for breeding programs that promote scrapie resistance. Breeding goats resistant to scrapie, while keeping valuable phenotypic traits, will help with eradication efforts and can be a valuable tool for goat producers and industry stakeholders. This information is particularly helpful for operations that need to bring in large numbers of does, which puts them at increased risk for scrapie introduction. Although PRNP genotypes have been previously characterized in the U.S. goat population [26], estimates of allele and genotype frequencies for the U.S. goat population have not been established. In this study, the genetic variability of eight codons 127, 143, 146, 154, 211, 222 and 240 in the PRNP gene locus and the differences in PRNP genotype frequencies were estimated in 6,029 goats representing 19 goat breeds. This is the first large-scale population study involving apparently scrapie disease-free goats from a large number of breeds and primary production type of operations in the U.S.

Similar to other studies, the genotypes that are associated with scrapie susceptibility (GG127, HH143, NN146, RR154 and QQ222) were predominant across all goats [14,22,26,41]. The relatively dominant homozygous genotypes over heterozygous genotypes may be due to inadequate crossbreeding and nonrandom mating practices among U.S. goat populations [42]. The wildtype allele S240 and its variant P240 are also commonly found across all goats and divided equally between the two allele sequences except in Oberhasli, Cashmere, and Toggenburg goats. A similar distribution of S240 and P240 has been reported previously in Italian [41], Cyprus gats [22,43], Moroccan [26], Norwegian white [44], and U.S. goats [26]. Association of the wild type alleles at codon 240 with scrapie resistance is weak because this codon is most likely excluded during the PrP post-translational processing inside the cell to generate the mature cellular protein and therefore cannot directly influence prion misfolding processes [15,21,24,41,45].

According to previous studies, allelic frequencies vary widely across populations. The S127, M142, R143, H154, S146, D146, H154, Q211, and K222 have been associated with scrapie resistance or a prolonged incubation period of scrapie in goats [4,24,25,46]. However, the resistant effect against scrapie was conferred by S146, D146, and K222 variants; and, therefore, they are considered the most ideal candidates for selective breeding programs in goats [25,47].

In this study, the resistant NS146 genotype was found in 22.0% [95%CI = (19.1, 25.2)] of goats across 68.6% [95%CI = (62.5, 74.1)] of operations. The NS146 was observed with varying frequencies across goat breeds. The NS146 genotype presented in more than 20% of Savannah, Boer, Nubian, LaMancha, Crossbred, and Cashmere goats and less than 5% of Alpine, Fainting, Saanen, Oberhasli, Nigerian, Pygmy, Angora, and Pygora goats. The NS146 genotype was most often observed in Savannah and Boer goat breeds, which have been mostly reared for meat production in the U.S. Similarly, the NS146 genotype was previously reported in Boer, Alpine, Fainting, LaMancha, Nubian, Pygmy, and Saanen goats from the U.S. and the UK [4,26]. This study added the Savannah, Cashmere, Kiko, Toggenburg, Nigerian dwarf, Spanish and Sable breeds to the list of known carriers of the resistant NS146 genotype. Another resistant genotype of the same codon ND146 was observed in 25.4% of Spanish goats [95%CI = (15.8, 38.2)], and in 1.2% or less of Boer, Cashmere, Angora, and Kiko goats. Akis et al., (2020) also reported a high frequency of the ND146 genotype in goats [42]. Comparisons between the goats from different primary operation production types showed a different distribution of the PRNP alleles with a higher percentage of the resistant genotypes in goats from meat operations compared to goats from dairy operations; which, was mainly driven by the observation of the NS146, ND146, and SS146 genotypes in higher percentages of Boer and Savannah goats. Resistant genotypes at 146 were the most widely distributed across operations, being present on 72.3% [95%CI = (66.3, 77.5)] of operations, and had the highest percentage of bucks with homozygous resistance, and therefore presents the greatest opportunity for building resistance in the US goat population.

In line with other studies, no homozygous KK222 goats were detected in this study [11,45]. The QK222 genotype was relatively rare and was estimated to be found in less than one percent of goats. Previous research in the U.S. reported the presence of K222 in only Toggenburg and LaMancha goats [26]. Our study added Alpine, Boer, Oberhasli, Saanen, Savannah, and Spanish breeds to the list of known carriers of the resistant K222 allele. Goats on operations in the east region had significantly higher percentages of the K222 allele, primarily driven by the fact that dairy operations are more frequently found in this region and the goat breeds that were observed with the highest percentages of the K222 allele were primarily dairy breeds (Oberhasli and Toggenburg). The low percentage of the QK222 genotype was also detected previously in goats, particularly in Saanen and Toggenburg goats [11]. In contrast, previous research in Italy reported higher frequencies of the K222 allele in goat breeds from southern Italy which may have occurred via breeding selection due to a higher prevalence of scrapie in goats in this region [48]. Several experimental challenge studies have examined the protective effect of the K222 allele against scrapie in goats. In the study of Acutis et al. (2012), all of the QK222 intracerebral challenged goats remained alive and scrapie disease free up to 4.5 years post inoculation [45]. Similarly, in the study of Lacroux et al. (2014), all QK222 heterozygous goats remained scrapie disease free up to approximately 6.8 years after scrapie oral inoculation [49]. Given the low percentage of the K222 allele in the U.S. goat population, breeding for scrapie resistance using the K222 allele will be challenging and therefore may require a stringent breeding strategy in breeds where the K222 allele is present. However, given the increased presence of the NS146 allele in a variety of goat breeds, it may be a promising allele to breed scrapie resistance into a higher number of U.S. goats. Alternatively, enhanced reproductive techniques, including artificial insemination could be an option in goat breeds that have the K222 allele present in low numbers to initially augment the number of animals with the protective allele. This could then be followed up with a more general breeding program to increase the resistance against scrapie.

Other alleles that were associated with increasing the scrapie incubation period (e.g., S127, M142, R143, H154, and Q211), previously reported in U.S. goats [26], were observed with varying frequencies in this study. Goats from the east region had a higher rate of RH154 and RQ211 genotypes. Similarly, the phylogeographic genetic structure has been previously demonstrated in goat breeds from different geographic regions [41,50,51]. In the study of Acutis et al., (2008), the genetic distance analysis revealed that the PRNP alleles of goats from northern locations differed than the goats from southern locations in Italy [41]. Similar differentiation between Italian goat breeds from northern and southern locations were obtained in a study that assessed PRNP genetic structure related to geographic distribution in European and Middle Eastern goat breeds using microsatellite and SNP markers [52]. Accordingly, the key determinants of the PRNP genetic variability are linked to the geographic origin of goat breeds combined with their population structure and the used breeding program that mainly depend on inbreeding strategies, that later results in genetic distinction of goat breeds [42,51,53].

Although not substantially different, the GS127 genotype was more common in Saanen and Sable goats compared with other goat breeds. According to a previous epidemiological study and in an experimental challenge, allele S127 appeared to give some protection against goat scrapie by delaying the appearance of clinical signs and increasing the incubation period when compared to wild-type goats [4,54]. A higher percentage of IM142 and MM142 genotypes were observed in Sable, Toggenburg, and Alpine goats, but these genotypes were absent in Cashmere, Pygmy, and Pygora goats. Similarly, the M142 variant had previously been found in Alpine, Toggenburg, Oberhasli, and LaMancha goats [26], and has been associated with lengthened scrapie incubation periods [14,21]. The mechanistic explanation of how the IM142 genotype plays a role in lengthening the incubation period of scrapie after experimental infection or increased scrapie resistance under natural conditions is still under investigation; however, it is likely related to the mutation in this allele that affects the conversion kinetics of PrP^C^ to PrP^Sc^ [44].

The R143 allele was detected in all examined goat breeds except Pygora and Sable goats. More than 20% each of Nigerian dwarf, Pygmy, Boer, and Savannah goats displayed the HR143 genotype while less than 5% each of Nubian, Toggenburg, Oberhasli, Saanen, Alpine, and Angora goats were HR143. Similarly, R143 allele had previously been found in goats located in Greece, the UK, and Japan and has been associated with resistance to scrapie [3,16,18]. In contrast, the study by Acutis et al. (2006) revealed no association of genotype HR143 with scrapie resistance in Italian goats [24]. This inconsistent result is therefore inadequate to support the role of the R143 allele in future breeding program to enhance scrapie resistance.

The RH154 genotype was found in a higher percentage of Fainting goats [16.6% with 95%CI = (6.3, 37.1)], Saanen [2.6% with 95%CI = (1.0, 6.8)], and Kiko goats [1.3% with 95%CI = (0.4, 3.9)] and less than 1.0% in all other specific, tested goat breeds. Similarly, RH154 genotype was detected at a high rate in both Afar and Arsi-Bale goat breeds [53]. Previous studies showed that the RH154 genotype confers an incomplete protection against scrapie in goats and showed similar frequencies in both scrapie-affected and healthy goats [22,24]. In contrast, the study by Lacroux et al. (2014) revealed that the RH154 genotype provide protection against scrapie after oral exposure [49]. The H154 allele is of particular interest because it is present in both the ovine and caprine PRNP gene and has been correlated with high susceptibility to the atypical scrapie in sheep, suggesting that it could be a risk factor for atypical scrapie in goats [14].

The RQ211 was present in most of the U.S. goat breeds with a higher rate in Sable, Oberhasli, Kiko, Alpine, LaMancha, Saanen, and Spanish goats and higher rates in goats from the east region. In other studies, the Q211 allele showed a different profile in goat breeds from different geographical locations with higher rate In Italian Pantellaria goats [42,55]. The RQ211 was also previously observed in U.S. goats [26], and has been associated with increasing the scrapie incubation period [22,43]. Similarly, epidemiological and experimental studies have indicated that the Q211 allele is associated with partial scrapie resistance, but scrapie positive cases in Q211 goats have been regularly found [56].

The most effective distribution of resistant genotypes throughout the goat population is through the mode of resistant bucks. From this study, it is estimated that 24.4% [95%CI = (17.4, 33.3)] of bucks accounting for just under a quarter of all operations [21.3% with 95%CI = (16.3, 27.3)] had bucks with resistant genotypes at codons 146 or 222, which represents a good baseline from which a growth of resistant bucks across U.S. goat operations could occur. Additionally, the results from this study suggest that a substantial portion of the overall differences in PRNP genetic diversity can be attributed to goat breeds, overall (10.0% of variability across allelic presentations across codons) and within State (5.6% of variability explained), after accounting for State, size, and primary production type of the operation, though there is still a substantial amount of residual variability between operations. In fact, the low intraclass correlation coefficients, ranging only from 9.8 to 23.9 across codons, and the relatively higher percentage of operations [72.8% with 95%CI = (66.9, 78.0)] with any goats with resistant genotypes (S146, D146, or K222) compared to the percentage of goats with resistant genotypes [33.8% with 95%CI = (29.6, 38.3)] supports this finding that resistant genotypes tend to occur at a shallow level on operations but more broadly across operations in the U.S.

Collectively, within the U.S. goat population there are goats with PRNP sequences that are associated with prolonged incubation periods and scrapie resistance, and the resistance associated alleles could be used to support breeding programs for enhanced scrapie resistance in goats reared in the U.S. It is therefore important to further investigate the caprine PRNP genetic variability using more herds naturally exposed to scrapie to clarify the association between PRNP genotypes and scrapie resistance or susceptibility. Additionally, further studies are needed to evaluate the protective level of PRNP genotypes in homozygous and heterozygous goats for an accurate assessment of breeding strategies to increase scrapie resistance in goats.

## Supporting information

S1 Table a. Percentage of goats by genotype at codon 127, by gender, region, primary production, and breed. b. Percentage of goats by genotype at codon 142, by gender, region, primary production, and breed. c. Percentage of goats by genotype at codon 143, by gender, region, primary production, and breed. d. Percentage of goats by genotype at codon 146, by gender, region, primary production, and breed. e. Percentage of goats by genotype at codon 154, by gender, region, primary production, and breed. f. Percentage of goats by genotype at codon 211, by gender, region, primary production, and breed. g. Percentage of goats by genotype at codon 222, by gender, region, primary production, and breed. h. Percentage of goats by genotype at codon 240, by gender, region, primary production, and breed. i. Percentage of goats by presence of S146, D146, or K222 genotypes, by gender, region, primary production, and breed.
(DOCX)

S2 Table a. Percentage of operations by genotype at codon 127, by region and primary production. b. Percentage of operations by genotype at codon 127, by region and primary production. c. Percentage of operations by genotype at codon 143, by region and primary production. d. Percentage of operations by genotype at codon 146, by region and primary production. e. Percentage of operations by genotype at codon 154, by region and primary production. f. Percentage of operations by genotype at codon 211, by region and primary production. g. Percentage of operations by genotype at codon 222, by region and primary production. h. Percentage of operations by genotype at codon 240, by region and primary production. i. Percentage of operations by presence of S146, D146, or K222 genotypes, by gender, region, primary production, and breed.
(DOCX)

## References

[1] USDA-NASS. United States Department of Agriculture-National Agricultural Statistics Service (USDA-NASS, 2020. Sheep and Goats (January 2020). ISSN:1949-1611. 2020.

[2] Stefania Thorgeirsdottir SS, Hjalti Mar Thorisson, Gudmundur Georgsson and Astridur Palsdottir. PrP gene polymorphism and natural scrapie in Icelandic sheep. Journal ofGeneral Virology. 1999;80:2527–34. doi: 10.1099/0022-1317-80-9-2527 10501510

[3] Charalambos Billinis CHP, Vassilios Psychas, Stamatis Argyroudis, Anna Nicolaou, Sotirios Leontides, Orestis Papadopoulos and Theodoros Sklaviadis. Important Prion protein gene polymorphisms in natural goat scrapie. Journal ofGeneral Virology. 2002;20:713–21. 10.1099/0022-1317-83-3-713 11842266

[4] Goldmann W, Ryan K, Stewart P, Parnham D, Xicohtencatl R, Fernandez N, et al. Caprine prion gene polymorphisms are associated with decreased incidence of classical scrapie in goat herds in the United Kingdom. Vet Res. 2011;42:110. Epub 2011/11/02. doi: 10.1186/1297-9716-42-110 ; PubMed Central PMCID: PMC3224758. 22040234 PMC3224758

[5] Eloit M, Adjou K, Coulpier M, Fontaine JJ, Hamel R, Lilin T, et al. BSE agent signatures in a goat. Veterinary Record. 2005;156(16):523–4. 10.1136/vr.156.16.523-b 15833975

[6] Hill AF, Butterworth RJ, Joiner S, Jackson G, Rossor MN, Thomas DJ, et al. Investigation of variant Creutzfeldt-Jakob disease and other human prion diseases with tonsil biopsy samples. The Lancet. 1999;353(9148):183–9. doi: 10.1016/s0140-6736(98)12075-5 9923873

[7] Wells GAH, Hawkins S.A.C., Green R.B., Austin A.R., Dexter I., Spencer Y.I., et al. Preliminary observations on the pathogenesis of experimental bovine spongiform encephalopathy (BSE): an update. Veterinary Record. 1998;142(5):103–6. doi: 10.1136/vr.142.5.103 9501384

[8] Katherine I. O’Rourke TEB, Miller M. W., Cline T. F., Spraker T. R., Jenny A. L., et al. PrP genotypes of captive and free-ranging Rocky Mountain elk. oonotics and Wildlife Disease. 1999;122. 10.1099/0022-1317-80-10-2765 10573173

[9] Greenlee JJ. Review: Update on Classical and Atypical Scrapie in Sheep and Goats. Vet Pathol. 2019;56(1):6–16. Epub 2018/09/12. doi: 10.1177/0300985818794247 . 30200819

[10] Prusiner SB. Prion Diseases and the BSE Crisis. SCIENCE. 1997;278. doi: 10.1126/science.278.5336.245 9323196

[11] Goldmann W, Marier E, Stewart P, Konold T, Street S, Langeveld J, et al. Prion protein genotype survey confirms low frequency of scrapie-resistant K222 allele in British goat herds. Vet Rec. 2016;178(7):168. Epub 2016/01/13. doi: 10.1136/vr.103521 ; PubMed Central PMCID: PMC4789823. 26755614 PMC4789823

[12] Foster JD, Parnham D., Chong A., Goldmann W. and Hunter N. Clinical signs, histopathology and genetics of experimental transmission of BSE and natural scrapie to sheep and goats. Veterinary Record. 2001;148(6):165–71. 10.1136/vr.148.6.165 11258721

[13] Hadlow WJ. The Pathology of Experimental Scrapie in the Dairy Goat. Research in Veterinary Science. 1961;2(4):289–329. doi: 10.1016/s0034-5288(18)34934-8

[14] Barillet F, Mariat D, Amigues Y, Faugeras R, Caillat H, Moazami-Goudarzi K, et al. Identification of seven haplotypes of the caprine PrP gene at codons 127, 142, 154, 211, 222 and 240 in French Alpine and Saanen breeds and their association with classical scrapie. J Gen Virol. 2009;90(Pt 3):769–76. Epub 2009/02/17. doi: 10.1099/vir.0.006114-0 . 19218225

[15] Corbiere F, Perrin-Chauvineau C, Lacroux C, Costes P, Thomas M, Bremaud I, et al. PrP-associated resistance to scrapie in five highly infected goat herds. J Gen Virol. 2013;94(Pt 1):241–5. Epub 2012/10/27. doi: 10.1099/vir.0.047225-0 . 23100359

[16] Goldmann W, Perucchini M., Smith A., and Hunter N.. Genetic variability of the PrP gene in a goat herd in the UK. Veteriniary Record. 2004:177–8. 10.1136/vr.155.6.177 15357379

[17] Papasavva-Stylianou P, Simmons MM, Ortiz-Pelaez A, Windl O, Spiropoulos J, Georgiadou S. Effect of Polymorphisms at Codon 146 of the Goat PRNP Gene on Susceptibility to Challenge with Classical Scrapie by Different Routes. J Virol. 2017;91(22). Epub 2017/09/08. doi: 10.1128/JVI.01142-17 ; PubMed Central PMCID: PMC5660499. 28878088 PMC5660499

[18] Yasuhisa KUROSAKI NI, Motohiro HORIUCHI and Morikazu SHINAGAWA. important Polymorphisms of prp gene in japan. J Vet Med Sci. 2005;67(3):321–3. doi: 10.1292/jvms.67.321 15805738

[19] Matsuura Y, Miyazawa K, Imamura M, Yokoyama T, Iwamaru Y. First case of atypical scrapie in a goat in Japan. J Vet Med Sci. 2019;81(7):986–9. Epub 2019/05/17. doi: 10.1292/jvms.18-0710 ; PubMed Central PMCID: PMC6656802. 31092762 PMC6656802

[20] Kim SK, Kim YC, Won SY, Jeong BH. Potential scrapie-associated polymorphisms of the prion protein gene (PRNP) in Korean native black goats. Sci Rep. 2019;9(1):15293. Epub 2019/10/28. doi: 10.1038/s41598-019-51621-y ; PubMed Central PMCID: PMC6814802. 31653880 PMC6814802

[21] Fast C, Goldmann W, Berthon P, Tauscher K, Andreoletti O, Lantier I, et al. Protecting effect of PrP codons M142 and K222 in goats orally challenged with bovine spongiform encephalopathy prions. Vet Res. 2017;48(1):52. Epub 2017/09/21. doi: 10.1186/s13567-017-0455-0 ; PubMed Central PMCID: PMC5606029. 28927447 PMC5606029

[22] Papasavva-Stylianou P, Kleanthous M, Toumazos P, Mavrikiou P, Loucaides P. Novel polymorphisms at codons 146 and 151 in the prion protein gene of Cyprus goats, and their association with natural scrapie. Vet J. 2007;173(2):459–62. Epub 2005/11/30. doi: 10.1016/j.tvjl.2005.09.013 . 16314132

[23] Srithayakumar V, Mitchell GB, White BN. Identification of amino acid variation in the prion protein associated with classical scrapie in Canadian dairy goats. BMC Vet Res. 2016;12:59. Epub 2016/03/24. doi: 10.1186/s12917-016-0684-x ; PubMed Central PMCID: PMC4804529. 27005313 PMC4804529

[24] Acutis PL, Bossers A, Priem J, Riina MV, Peletto S, Mazza M, et al. Identification of prion protein gene polymorphisms in goats from Italian scrapie outbreaks. J Gen Virol. 2006;87(Pt 4):1029–33. Epub 2006/03/11. doi: 10.1099/vir.0.81440-0 . 16528054

[25] White SN, Reynolds JO, Waldron DF, Schneider DA, O’Rourke KI. Extended scrapie incubation time in goats singly heterozygous for PRNP S146 or K222. Gene. 2012;501(1):49–51. Epub 2012/04/21. doi: 10.1016/j.gene.2012.03.068 . 22516690

[26] White S, Herrmann-Hoesing L, O’Rourke K, Waldron D, Rowe J, Alverson J. Prion gene (PRNP) haplotype variation in United States goat breeds (Open Access publication). Genet Sel Evol. 2008;40(5):553–61. Epub 2008/08/13. doi: 10.1186/1297-9686-40-5-553 ; PubMed Central PMCID: PMC2674890. 18694550 PMC2674890

[27] Mazza M, Guglielmetti C, Ingravalle F, Brusadore S, Langeveld JP, Ekateriniadou LV, et al. Low fraction of the 222K PrP variant in the protease-resistant moiety of PrPres in heterozygous scrapie positive goats. The Journal of general virology. 2017;98(7):1963. doi: 10.1099/jgv.0.000843 28691895 PMC5656779

[28] USDA-APHIS. United States Department of Agriculture-Animal and Plant Health Inspection Service (USDA-APHIS), 2020. Goat 2019 –Part I: Reference of Goat Management Practices in the United States, 2019. Unpublished. 2020.

[29] Särndal C-E, Bengt Swensson, and Jan Wretman. Model assisted survey sampling: Springer Science & Business Media, 2003.; 2003.

[30] Wald A. Tests of statistical hypotheses concerning several parameters when the number of observations is large. Transactions of the American Mathematical society. 1943;54(3):426–82.

[31] Fellegi IP. Approximate tests of independence and goodness of fit based on stratified multistage samples. Journal of the American Statistical Association. 1980;75(370):261–8.

[32] Fleiss JL, Cuzick J. The reliability of dichotomous judgments: Unequal numbers of judges per subject. Applied Psychological Measurement. 1979;3(4):537–42.

[33] Excoffier L, Smouse PE, Quattro JM. Analysis of molecular variance inferred from metric distances among DNA haplotypes: application to human mitochondrial DNA restriction data. Genetics. 1992;131(2):479–91. 1644282 10.1093/genetics/131.2.479 PMC1205020

[34] Nei M. Genetic distance between populations. The American Naturalist. 1972;106(949):283–92.

[35] Anderson MJ, Walsh DC. PERMANOVA, ANOSIM, and the Mantel test in the face of heterogeneous dispersions: what null hypothesis are you testing? Ecological monographs. 2013;83(4):557–74.

[36] Anderson MJ. Distance‐based tests for homogeneity of multivariate dispersions. Biometrics. 2006;62(1):245–53. doi: 10.1111/j.1541-0420.2005.00440.x 16542252

[37] Johnson RA, Wichern DW. Applied multivariate statistical analysis: Prentice hall Upper Saddle River, NJ; 2002.

[38] Dray S, Dufour A-B. The ade4 package: implementing the duality diagram for ecologists. Journal of statistical software. 2007;22(4):1–20.

[39] APHIS U. USDA APHIS: National Scrapie Eradication Program. Available at: wwwaphisusdagov/aphis/ourfocus/animalhealth/animal-disease-information/sheep-and-goat-health/national-scrapie-eradication-program. 2020:Accessed Feb 11, 2020.

[40] Greenlee JJ. Update on classical and atypical scrapie in sheep and goats. Veterinary pathology. 2019;56(1):6–16. doi: 10.1177/0300985818794247 30200819

[41] Acutis PL, Colussi S, Santagada G, Laurenza C, Maniaci MG, Riina MV, et al. Genetic variability of the PRNP gene in goat breeds from Northern and Southern Italy. J Appl Microbiol. 2008;104(6):1782–9. Epub 2008/01/26. doi: 10.1111/j.1365-2672.2007.03703.x . 18217941

[42] Akis I, Oztabak K, Atmaca G, Esen Gursel F, Ates A, Yardibi H, et al. PRNP gene polymorphisms in main indigenous Turkish goat breeds. Trop Anim Health Prod. 2020;52(2):793–802. Epub 2019/10/21. doi: 10.1007/s11250-019-02070-2 . 31630310

[43] Papasavva-Stylianou P, Windl O, Saunders G, Mavrikiou P, Toumazos P, Kakoyiannis C. PrP gene polymorphisms in Cyprus goats and their association with resistance or susceptibility to natural scrapie. Vet J. 2011;187(2):245–50. Epub 2010/01/23. doi: 10.1016/j.tvjl.2009.10.015 . 20093056

[44] Kipanyula MJ, Chuma IS, Brundtland E, Bårdsen K, Msalya G, Kifaro GC, et al. Prion protein (PrP) gene polymorphisms in Small East African and Norwegian white goats. Small Ruminant Research. 2014;121(2–3):200–6. doi: 10.1016/j.smallrumres.2014.06.002

[45] Acutis PL, Martucci F., D’Angelo A., Peletto S., Colussi S., Maurella C., et al, Resistance to classical scrapie in experimentally challenged goats carrying mutation K222 of the prion protein gene. Veterinary Research. 2012;32:8. doi: 10.1186/1297-9716-43-8 22296670 PMC3296670

[46] Zhou RY, Li XL, Li LH, Wang HY, Lu JG. Polymorphism of the PRNP gene in the main breeds of indigenous Chinese goats. Arch Virol. 2008;153(5):979–82. Epub 2008/03/29. doi: 10.1007/s00705-008-0074-1 . 18369524

[47] Cinar MU, Schneider DA, Waldron DF, O’Rourke KI, White SN. Goats singly heterozygous for PRNP S146 or K222 orally inoculated with classical scrapie at birth show no disease at ages well beyond 6 years. Vet J. 2018;233:19–24. Epub 2018/03/01. doi: 10.1016/j.tvjl.2017.12.019 . 29486874

[48] Windig JJ, Hoving RA, Priem J, Bossers A, van Keulen LJ, Langeveld JP. Variation in the prion protein sequence in Dutch goat breeds. J Anim Breed Genet. 2016;133(5):366–74. Epub 2016/03/19. doi: 10.1111/jbg.12211 . 26991480

[49] Lacroux C, Perrin-Chauvineau C, Corbiere F, Aron N, Aguilar-Calvo P, Torres JM, et al. Genetic resistance to scrapie infection in experimentally challenged goats. J Virol. 2014;88(5):2406–13. Epub 2013/11/29. doi: 10.1128/JVI.02872-13 ; PubMed Central PMCID: PMC3958109. 24284317 PMC3958109

[50] Vouraki S, Gelasakis AI, Alexandri P, Boukouvala E, Ekateriniadou LV, Banos G, et al. Genetic profile of scrapie codons 146, 211 and 222 in the PRNP gene locus in three breeds of dairy goats. PLoS One. 2018;13(6):e0198819. Epub 2018/06/08. doi: 10.1371/journal.pone.0198819 ; PubMed Central PMCID: PMC5991713. 29879210 PMC5991713

[51] Nonno R, Marin-Moreno A, Carlos Espinosa J, Fast C, Van Keulen L, Spiropoulos J, et al. Characterization of goat prions demonstrates geographical variation of scrapie strains in Europe and reveals the composite nature of prion strains. Sci Rep. 2020;10(1):19. Epub 2020/01/09. doi: 10.1038/s41598-019-57005-6 ; PubMed Central PMCID: PMC6949283. 31913327 PMC6949283

[52] Pariset L, Cappuccio I, Ajmone Marsan P, Dunner S, Luikart G, England PR, et al. Assessment of population structure by single nucleotide polymorphisms (SNPs) in goat breeds. J Chromatogr B Analyt Technol Biomed Life Sci. 2006;833(1):117–20. Epub 2006/02/14. doi: 10.1016/j.jchromb.2006.01.011 . 16473052

[53] Teferedegn EY, Yaman Y, Un C. Novel Variations in Native Ethiopian Goat breeds PRNP Gene and Their Potential Effect on Prion Protein Stability. Sci Rep. 2020;10(1):6953. Epub 2020/04/26. doi: 10.1038/s41598-020-63874-z ; PubMed Central PMCID: PMC7181617. 32332800 PMC7181617

[54] Dassanayake RP, White SN, Madsen-Bouterse SA, Schneider DA, O’Rourke KI. Role of the PRNP S127 allele in experimental infection of goats with classical caprine scrapie. Anim Genet. 2015;46(3):341. Epub 2015/04/29. doi: 10.1111/age.12291 ; PubMed Central PMCID: PMC5132141. 25917307 PMC5132141

[55] Migliore S, Agnello S, Chiappini B, Vaccari G, Mignacca SA, Di Marco Lo Presti V, et al. Biodiversity and selection for scrapie resistance in goats: Genetic polymorphism in “Girgentana” breed in Sicily, Italy. Small Ruminant Research. 2015;125:137–41. doi: 10.1016/j.smallrumres.2015.01.029

[56] Ricci A, Allende A, Bolton D, Chemaly M, Davies R, Fernandez Escamez PS, et al. Genetic resistance to transmissible spongiform encephalopathies (TSE) in goats. EFSA J. 2017;15(8):e04962. Epub 2017/08/10. doi: 10.2903/j.efsa.2017.4962 ; PubMed Central PMCID: PMC7010077. 32625625 PMC7010077

