# Supplementary material for: Large-scale survey of prion protein genetic variability in scrapie disease-free goats from the United States
Source: PLoS One. 2021 Jul 19;16(7):e0254998. doi: 10.1371/journal.pone.0254998 (PMC8289333; doi:10.1371/journal.pone.0254998)
Supplement: S1 Table — a. Percentage of goats by genotype at codon 127, by gender, region, primary production, and breed. b. Percentage of goats by genotype at codon 142, by gender, region, primary production, and breed. c. Percentage of goats by genotype at codon 143, by gender, region, primary production, and breed. d. Percentage of goats by genotype at codon 146, by gender, region, primary production, and breed. e. Percentage of goats by genotype at codon 154, by gender, region, primary production, and breed. f. Percentage of goats by genotype at codon 211, by gender, region, primary production, and breed. g. Percentage of goats by genotype at codon 222, by gender, region, primary production, and breed. h. Percentage of goats by genotype at codon 240, by gender, region, primary production, and breed. i. Percentage of goats by presence of S146, D146, or K222 genotypes, by gender, region, primary production, and breed. (DOCX) [file pone.0254998.s001.docx]

Supplementary materials

# Percentage of goats by codon, by genotype, and by breakout variables

**Table S1.a.** Percentage of goats by genotype at codon 127, by gender, region, primary production, and breed.

|  | Percent goats | | | | | | | | |
| --- | --- | --- | --- | --- | --- | --- | --- | --- | --- |
|  | Genotype | | | | | | | | |
|  | GG | | GS | | SS | | Total | GS or SS | |
| Breakout variable | Pct | 95% CI | Pct | 95% CI | Pct | 95% CI | Pct | Pct | 95% CI |
| Overall | 99.7 | (99.5,99.8) | 0.3 | (0.2,0.5) | 0.0 | (0.0,0.0) | 100.0 | 0.3 | (0.2,0.5) |
| Gender | | | | | | | |  |  |
| Doe | 99.7 | (99.4,99.8) | 0.3 | (0.2,0.6) | 0.0 | (–) | 100.0 | 0.3 | (0.2,0.6) |
| Buck | 99.9 | (99.8,100.0) | 0.1 | (0.0,0.2) | 0.0 | (0.0,0.0) | 100.0 | 0.1 | (0.0,0.2) |
| Region | | | | | | | |  |  |
| West | 99.7 | (99.1,99.9) | 0.3 | (0.1,0.9) | 0.0 | (–) | 100.0 | 0.3 | (0.1,0.9) |
| East | 99.7 | (99.5,99.8) | 0.3 | (0.2,0.5) | 0.0 | (0.0,0.0) | 100.0 | 0.3 | (0.2,0.5) |
| Primary production | | | | | | | |  |  |
| Meat | 100.0 | (99.7,100.0) | 0.0 | (0.0,0.3) | 0.0 | (–) | 100.0 | 0.0 | (0.0,0.3) |
| Dairy | 99.2 | (98.2,99.6) | 0.8 | (0.4,1.8) | 0.0 | (0.0,0.0) | 100.0 | 0.8 | (0.4,1.8) |
| Other | 99.6 | (98.8,99.8) | 0.4 | (0.2,1.2) | 0.0 | (–) | 100.0 | 0.4 | (0.2,1.2) |
| Breed | | | | | | | |  |  |
| Alpine | 100.0 | (–) | 0.0 | (–) | 0.0 | (–) | 100.0 | 0.0 | (–) |
| Angora | 100.0 | (–) | 0.0 | (–) | 0.0 | (–) | 100.0 | 0.0 | (–) |
| Boer | 99.9 | (99.4,100) | 0.1 | (0.0,0.6) | 0.0 | (–) | 100.0 | 0.1 | (0.0,0.6) |
| Cashmere | 100.0 | (–) | 0.0 | (–) | 0.0 | (–) | 100.0 | 0.0 | (–) |
| Fainting goats | 100.0 | (–) | 0.0 | (–) | 0.0 | (–) | 100.0 | 0.0 | (–) |
| Kiko | 100.0 | (–) | 0.0 | (–) | 0.0 | (–) | 100.0 | 0.0 | (–) |
| LaMancha | 99.9 | (99.5,100) | 0.1 | (0.0,0.5) | 0.0 | (–) | 100.0 | 0.1 | (0.0,0.5) |
| Nigerian dwarf | 100.0 | (–) | 0.0 | (–) | 0.0 | (–) | 100.0 | 0.0 | (–) |
| Nubian | 99.8 | (98.8,100) | 0.2 | (0,1.2) | 0.0 | (–) | 100.0 | 0.2 | (0.0,1.2) |
| Oberhasli | 100.0 | (–) | 0.0 | (–) | 0.0 | (–) | 100.0 | 0.0 | (–) |
| Pygmy | 100.0 | (–) | 0.0 | (–) | 0.0 | (–) | 100.0 | 0.0 | (–) |
| Pygora | 100.0 | (–) | 0.0 | (–) | 0.0 | (–) | 100.0 | 0.0 | (–) |
| Saanen | 98.3 | (96.8,99.1) | 1.7 | (0.9,3.2) | 0.0 | (0.0,0.0) | 100.0 | 1.7 | (0.9,3.2) |
| Sable | 99.5 | (96.4,99.9) | 0.5 | (0.1,3.6) | 0.0 | (–) | 100.0 | 0.5 | (0.1,3.6) |
| Savannah | 100.0 | (–) | 0.0 | (–) | 0.0 | (–) | 100.0 | 0.0 | (–) |
| Spanish | 100.0 | (–) | 0.0 | (–) | 0.0 | (–) | 100.0 | 0.0 | (–) |
| Toggenburg | 100.0 | (–) | 0.0 | (–) | 0.0 | (–) | 100.0 | 0.0 | (–) |
| Crossbred | 99.1 | (97.8,99.6) | 0.9 | (0.4,2.2) | 0.0 | (–) | 100.0 | 0.9 | (0.4,2.2) |
| Other | 100.0 | (–) | 0.0 | (–) | 0.0 | (–) | 100.0 | 0.0 | (–) |

**Table S1.b.** Percentage of goats by genotype at codon 142, by gender, region, primary production, and breed.

|  | Percent goats | | | | | | | | |
| --- | --- | --- | --- | --- | --- | --- | --- | --- | --- |
|  | Genotype | | | | | | | | |
|  | II | | IM | | MM | | Total | IM or MM | |
| Breakout variable | Pct | 95% CI | Pct | 95% CI | Pct | 95% CI | Pct | Pct | 95% CI |
| Overall | 91.9 | (89.5,93.8) | 7.5 | (5.8,9.7) | 0.6 | (0.3,1.1) | 100.0 | 8.1 | (6.2,10.5) |
| Gender | | | | | | | |  |  |
| Doe | 91.6 | (89.1,93.6) | 7.8 | (5.9,10.1) | 0.6 | (0.3,1.2) | 100.0 | 8.4 | (6.4,10.9) |
| Buck | 94.9 | (91.2,97.0) | 5.1 | (2.9,8.7) | 0.1 | (0.0,0.2) | 100.0 | 5.1 | (3.0,8.8) |
| Region | | | | | | | |  |  |
| West | 93.4 | (89.4,96.0) | 6.5 | (3.9,10.5) | 0.1 | (0.0,0.4) | 100.0 | 6.6 | (4,10.6) |
| East | 90.7 | (87.3,93.2) | 8.4 | (6.2,11.2) | 0.9 | (0.4,1.9) | 100.0 | 9.3 | (6.8,12.7) |
| Primary production | | | | | | | |  |  |
| Meat | 94.8 | (91.3,96.9) | 5.0 | (3.0,8.4) | 0.2 | (0.0,0.9) | 100.0 | 5.2 | (3.1,8.7) |
| Dairy | 79.4 | (72.5,84.9) | 18.4 | (13.8,24.0) | 2.3 | (1.1,4.7) | 100.0 | 20.6 | (15.1,27.5) |
| Other | 95.2 | (92.3,97.1) | 4.7 | (2.8,7.6) | 0.1 | (0.0,0.3) | 100.0 | 4.8 | (2.9,7.7) |
| Breed | | | | | | | |  |  |
| Alpine | 61.8 | (43.7,77.1) | 31.0 | (19.6,45.2) | 7.2 | (3.3,15.1) | 100.0 | 38.2 | (22.9,56.3) |
| Angora | 98.8 | (92.0,99.8) | 1.2 | (0.2,8.0) | 0.0 | (–) | 100.0 | 1.2 | (0.2,8.0) |
| Boer | 96.5 | (93.3,98.2) | 3.2 | (1.8,5.9) | 0.3 | (0.0,2.0) | 100.0 | 3.5 | (1.8,6.7) |
| Cashmere | 100.0 | (–) | 0.0 | (–) | 0.0 | (–) | 100.0 | 0.0 | (–) |
| Fainting goats | 92.3 | (69.3,98.5) | 7.4 | (1.4,30.9) | 0.3 | (0.0,2.3) | 100.0 | 7.7 | (1.5,30.7) |
| Kiko | 95.1 | (89.2,97.9) | 4.9 | (2.1,10.8) | 0.0 | (–) | 100.0 | 4.9 | (2.1,10.8) |
| LaMancha | 86.5 | (70.3,94.5) | 13.5 | (5.4,29.7) | 0.0 | (0.0,0.0) | 100.0 | 13.5 | (5.5,29.7) |
| Nigerian dwarf | 98.7 | (96.4,99.5) | 1.3 | (0.5,3.6) | 0.0 | (–) | 100.0 | 1.3 | (0.5,3.6) |
| Nubian | 89.4 | (80.8,94.4) | 10.5 | (5.5,19.1) | 0.1 | (0.0,0.9) | 100.0 | 10.6 | (5.6,19.2) |
| Oberhasli | 87.2 | (73.4,94.4) | 12.8 | (5.6,26.6) | 0.0 | (–) | 100.0 | 12.8 | (5.6,26.6) |
| Pygmy | 100.0 | (–) | 0.0 | (–) | 0.0 | (–) | 100.0 | 0.0 | (–) |
| Pygora | 100.0 | (–) | 0.0 | (–) | 0.0 | (–) | 100.0 | 0.0 | (–) |
| Saanen | 86.2 | (78.1,91.7) | 13.7 | (8.2,21.9) | 0.1 | (0.0,0.6) | 100.0 | 13.8 | (8.3,21.9) |
| Sable | 43.6 | (9.8,84.7) | 54.9 | (14.2,90.0) | 1.5 | (0.2,10.8) | 100.0 | 56.4 | (15.3,90.2) |
| Savannah | 84.1 | (52.5,96.2) | 15.3 | (3.5,47.6) | 0.7 | (0.1,5.4) | 100.0 | 15.9 | (3.8,47.5) |
| Spanish | 94.0 | (83.2,98.0) | 6.0 | (2.0,16.8) | 0.0 | (–) | 100.0 | 6.0 | (2.0,16.8) |
| Toggenburg | 32.8 | (13.0,61.3) | 61.9 | (33.3,84.1) | 5.3 | (1.8,14.3) | 100.0 | 67.2 | (38.7,87) |
| Crossbred | 90.3 | (87.1,92.8) | 9.2 | (6.8,12.4) | 0.5 | (0.2,1.1) | 100.0 | 9.7 | (7.2,12.9) |
| Other | 91.4 | (78.3,96.9) | 7.9 | (2.7,21.0) | 0.7 | (0.1,4.6) | 100.0 | 8.6 | (3.1,21.7) |

**Table S1.c.** Percentage of goats by genotype at codon 143, by gender, region, primary production, and breed.

|  | Percent goats | | | | | | | | |
| --- | --- | --- | --- | --- | --- | --- | --- | --- | --- |
|  | Genotype | | | | | | | | |
|  | HH | | HR | | RR | | Total | HR or RR | |
| Breakout variable | Pct | 95% CI | Pct | 95% CI | Pct | 95% CI | Pct | Pct | 95% CI |
| Overall | 85.2 | (82.9,87.3) | 13.7 | (11.8,15.9) | 1.1 | (0.6,1.8) | 100.0 | 14.8 | (12.7,17.1) |
| Gender | | | | | | | |  |  |
| Doe | 85.3 | (82.8,87.5) | 13.9 | (11.8,16.2) | 0.8 | (0.5,1.5) | 100.0 | 14.7 | (12.5,17.2) |
| Buck | 84.2 | (77.4,89.2) | 12.2 | (8.4,17.4) | 3.6 | (1.2,10.7) | 100.0 | 15.8 | (10.8,22.6) |
| Region | | | | | | | |  |  |
| West | 85.2 | (81.2,88.4) | 13.7 | (10.6,17.5) | 1.1 | (0.5,2.5) | 100.0 | 14.8 | (11.6,18.8) |
| East | 85.2 | (82.3,87.8) | 13.7 | (11.4,16.4) | 1.0 | (0.5,2.1) | 100.0 | 14.8 | (12.2,17.7) |
| Primary production | | | | | | | |  |  |
| Meat | 82.1 | (78.7,85) | 16.9 | (14.0,20.1) | 1.1 | (0.5,2.1) | 100.0 | 17.9 | (15,21.3) |
| Dairy | 93.3 | (90.8,95.2) | 6.1 | (4.4,8.3) | 0.6 | (0.2,1.5) | 100.0 | 6.7 | (4.8,9.2) |
| Other | 86.1 | (79.9,90.6) | 12.4 | (8.6,17.5) | 1.5 | (0.5,4.3) | 100.0 | 13.9 | (9.4,20.1) |
| Breed | | | | | | | |  |  |
| Alpine | 99.9 | (99.5,100.0) | 0.1 | (0.0,0.5) | 0.0 | (–) | 100.0 | 0.1 | (0.0,0.5) |
| Angora | 99.9 | (99.5,100.0) | 0.1 | (0.0,0.5) | 0.0 | (–) | 100.0 | 0.1 | (0.0,0.5) |
| Boer | 73.4 | (68.9,77.5) | 23.9 | (20.3,27.8) | 2.7 | (1.3,5.3) | 100.0 | 26.6 | (22.5,31.1) |
| Cashmere | 82.9 | (52.5,95.5) | 9.9 | (2.9,29.2) | 7.2 | (1.5,27.8) | 100.0 | 17.1 | (4.5,47.5) |
| Fainting goats | 100.0 | (99.7,100.0) | 0.0 | (0.0,0.3) | 0.0 | (–) | 100.0 | 0.0 | (0.0,0.3) |
| Kiko | 82.0 | (69.8,90.0) | 16.6 | (8.9,29.0) | 1.4 | (0.5,4.2) | 100.0 | 18.0 | (10.0,30.2) |
| LaMancha | 100.0 | (99.9,100.0) | 0.0 | (0.0,0.1) | 0.0 | (–) | 100.0 | 0.0 | (0.0,0.1) |
| Nigerian dwarf | 67.1 | (58.1,75.0) | 27.9 | (21.4,35.4) | 5.0 | (2.0,11.9) | 100.0 | 32.9 | (25.0,41.9) |
| Nubian | 95.3 | (90.0,97.8) | 4.6 | (2.1,9.8) | 0.1 | (0.0,0.8) | 100.0 | 4.7 | (2.2,10.0) |
| Oberhasli | 99.6 | (97.5,99.9) | 0.4 | (0.1,2.5) | 0.0 | (–) | 100.0 | 0.4 | (0.1,2.5) |
| Pygmy | 73.0 | (57.8,84.3) | 27.0 | (15.7,42.2) | 0.0 | (–) | 100.0 | 27.0 | (15.7,42.2) |
| Pygora | 100.0 | (–) | 0.0 | (–) | 0.0 | (–) | 100.0 | 0.0 | (–) |
| Saanen | 99.6 | (97.2,99.9) | 0.4 | (0.1,2.8) | 0.0 | (–) | 100.0 | 0.4 | (0.1,2.8) |
| Sable | 100.0 | (–) | 0.0 | (–) | 0.0 | (–) | 100.0 | 0.0 | (–) |
| Savannah | 79.1 | (56.4,91.8) | 20.9 | (8.2,43.6) | 0.0 | (–) | 100.0 | 20.9 | (8.2,43.6) |
| Spanish | 88.4 | (82.3,92.6) | 11.5 | (7.4,17.6) | 0.1 | (0.0,0.4) | 100.0 | 11.6 | (7.4,17.7) |
| Toggenburg | 99.4 | (96.2,99.9) | 0.6 | (0.1,3.8) | 0.0 | (–) | 100.0 | 0.6 | (0.1,3.8) |
| Crossbred | 87.5 | (83.5,90.6) | 12.1 | (9.0,16.1) | 0.4 | (0.1,1.1) | 100.0 | 12.5 | (9.4,16.5) |
| Other | 92.9 | (81.2,97.6) | 6.1 | (2.0,17.1) | 0.9 | (0.1,6.4) | 100.0 | 7.1 | (2.4,18.8) |

**Table S1.d.** Percentage of goats by genotype at codon 146, by gender, region, primary production, and breed.

|  | Percent goats | | | | | | | | | | | | | | |
| --- | --- | --- | --- | --- | --- | --- | --- | --- | --- | --- | --- | --- | --- | --- | --- |
|  | Genotype | | | | | | | | | | | | | | |
|  | NN | | NS | | ND | | SS | | DD | | SD | | Total | Any non-NN | |
| Breakout variable | Pct | 95% CI | Pct | 95% CI | Pct | 95% CI | Pct | 95% CI | Pct | 95% CI | Pct | 95% CI | Pct | Pct | 95% CI |
| Overall | 66.7 | (62.2,71.0) | 22.0 | (19.1,25.2) | 6.3 | (3.6,10.7) | 4.0 | (2.9,5.5) | 0.7 | (0.2,2.3) | 0.3 | (0.1,1.1) | 100.0 | 33.3 | (29.0,37.8) |
| Gender | | | | | | | | | | | | | |  |  |
| Doe | 65.8 | (61.1,70.3) | 22.3 | (19.3,25.7) | 6.7 | (3.8,11.5) | 4.1 | (2.9,5.8) | 0.8 | (0.2,2.5) | 0.3 | (0.1,1.2) | 100.0 | 34.2 | (29.7,38.9) |
| Buck | 76.4 | (67.6,83.3) | 19.0 | (12.6,27.5) | 1.7 | (0.6,4.5) | 2.9 | (1.6,5.0) | 0.0 | (–) | 0.1 | (0.0,0.8) | 100.0 | 23.6 | (16.7,32.4) |
| Region | | | | | | | | | | | | | |  |  |
| West | 62.4 | (54.5,69.7) | 23.3 | (19.0,28.4) | 8.1 | (3.8,16.3) | 4.3 | (2.6,7.1) | 1.4 | (0.4,4.9) | 0.5 | (0.1,2.5) | 100.0 | 37.6 | (30.3,45.5) |
| East | 70.3 | (65.5,74.7) | 21.0 | (17.2,25.3) | 4.8 | (2.2,10.4) | 3.8 | (2.4,5.7) | 0.1 | (0.0,0.3) | 0.1 | (0.0,0.3) | 100.0 | 29.7 | (25.3,34.5) |
| Primary production | | | | | | | | | | | | | |  |  |
| Meat | 58.9 | (53.3,64.2) | 24.9 | (21.0,29.3) | 9.2 | (5.3,15.6) | 5.4 | (3.8,7.7) | 1.1 | (0.3,3.9) | 0.4 | (0.1,1.9) | 100.0 | 41.1 | (35.8,46.7) |
| Dairy | 82.9 | (78.1,86.8) | 15.5 | (11.7,20.1) | 0.4 | (0.1,1.4) | 1.2 | (0.7,2.0) | 0.0 | (–) | 0.1 | (0.0,1.0) | 100.0 | 17.1 | (13.2,21.9) |
| Other | 72.5 | (61.8,81.2) | 20.4 | (13.9,29.0) | 3.9 | (0.7,19.3) | 2.9 | (1.1,7.7) | 0.2 | (0.0,1.3) | 0.1 | (0.0,0.3) | 100.0 | 27.5 | (18.8,38.2) |
| Breed | | | | | | | | | | | | | |  |  |
| Alpine | 96.2 | (90.6,98.5) | 3.8 | (1.5,9.4) | 0.0 | (–) | 0.0 | (0.0,0.3) | 0.0 | (–) | 0.0 | (–) | 100.0 | 3.8 | (1.5,9.4) |
| Angora | 99.1 | (93.5,99.9) | 0.0 | (–) | 0.9 | (0.1,6.5) | 0.0 | (–) | 0.0 | (–) | 0.0 | (–) | 100.0 | 0.9 | (0.1,6.5) |
| Boer | 53.0 | (48.9,57.1) | 36.7 | (33.1,40.4) | 1.2 | (0.5,3.3) | 8.9 | (6.4,12.5) | 0.0 | (–) | 0.1 | (0.0,0.7) | 100.0 | 47.0 | (42.9,51.1) |
| Cashmere | 77.0 | (40.1,94.4) | 21.8 | (5.0,59.3) | 1.2 | (0.1,10.0) | 0.0 | (–) | 0.0 | (–) | 0.0 | (–) | 100.0 | 23.0 | (5.6,59.9) |
| Fainting goats | 96.6 | (83.9,99.4) | 3.4 | (0.6,16.1) | 0.0 | (–) | 0.0 | (–) | 0.0 | (–) | 0.0 | (–) | 100.0 | 3.4 | (0.6,16.1) |
| Kiko | 82.8 | (66.8,92.0) | 16.3 | (7.3,32.7) | 0.5 | (0.1,2.9) | 0.0 | (–) | 0.0 | (–) | 0.4 | (0.1,2.6) | 100.0 | 17.2 | (8.0,33.2) |
| LaMancha | 64.2 | (49.0,77.0) | 35.6 | (22.8,50.8) | 0.0 | (–) | 0.2 | (0.1,0.7) | 0.0 | (–) | 0.0 | (–) | 100.0 | 35.8 | (23.0,51.0) |
| Nigerian dwarf | 99.3 | (97.9,99.8) | 0.7 | (0.2,2.1) | 0.0 | (–) | 0.0 | (–) | 0.0 | (–) | 0.0 | (–) | 100.0 | 0.7 | (0.2,2.1) |
| Nubian | 61.4 | (51.8,70.2) | 36.3 | (27.0,46.7) | 0.0 | (0.0,0.2) | 2.3 | (1.1,5.0) | 0.0 | (–) | 0.0 | (–) | 100.0 | 38.6 | (29.8,48.2) |
| Oberhasli | 98.3 | (92.0,99.6) | 1.7 | (0.4,8.0) | 0.0 | (–) | 0.0 | (–) | 0.0 | (–) | 0.0 | (–) | 100.0 | 1.7 | (0.4,8.0) |
| Pygmy | 99.4 | (96.6,99.9) | 0.6 | (0.1,3.4) | 0.0 | (–) | 0.0 | (–) | 0.0 | (–) | 0.0 | (–) | 100.0 | 0.6 | (0.1,3.4) |
| Pygora | 98.9 | (89.9,99.9) | 0.0 | (–) | 0.0 | (–) | 1.1 | (0.1,10.1) | 0.0 | (–) | 0.0 | (–) | 100.0 | 1.1 | (0.1,10.1) |
| Saanen | 97.2 | (92.6,99.0) | 2.8 | (1.0,7.4) | 0.0 | (–) | 0.0 | (–) | 0.0 | (–) | 0.0 | (–) | 100.0 | 2.8 | (1.0,7.4) |
| Sable | 91.5 | (52.3,99.1) | 8.5 | (0.9,47.7) | 0.0 | (–) | 0.0 | (–) | 0.0 | (–) | 0.0 | (–) | 100.0 | 8.5 | (0.9,47.7) |
| Savannah | 34.5 | (11.0,69.2) | 65.5 | (30.8,89.0) | 0.0 | (–) | 0.0 | (–) | 0.0 | (–) | 0.0 | (–) | 100.0 | 65.5 | (30.8,89.0) |
| Spanish | 57.4 | (43.9,69.9) | 11.4 | (6.7,18.7) | 25.4 | (15.8,38.2) | 1.9 | (0.5,7.1) | 2.9 | (0.9,9.3) | 1.0 | (0.2,5.4) | 100.0 | 42.6 | (30.1,56.1) |
| Toggenburg | 87.0 | (55.9,97.2) | 13.0 | (2.8,44.1) | 0.0 | (–) | 0.0 | (–) | 0.0 | (–) | 0.0 | (–) | 100.0 | 13.0 | (2.8,44.1) |
| Crossbred | 62.3 | (55.8,68.4) | 28.8 | (22.5,36.1) | 3.3 | (1.6,6.7) | 4.9 | (2.3,10.0) | 0.4 | (0.1,1.4) | 0.3 | (0.1,0.8) | 100.0 | 37.7 | (31.6,44.2) |
| Other | 87.7 | (75.2,94.4) | 11.6 | (5.1,24.3) | 0.6 | (0.1,4.6) | 0.0 | (–) | 0.0 | (–) | 0.0 | (–) | 100.0 | 12.3 | (5.6,24.8) |

**Table S1.e.** Percentage of goats by genotype at codon 154, by gender, region, primary production, and breed.

|  | Percent goats | | | | | | | | |
| --- | --- | --- | --- | --- | --- | --- | --- | --- | --- |
|  | Genotype | | | | | | | | |
|  | RR | | RH | | HH | | Total | RH or HH | |
| Breakout variable | Pct | 95% CI | Pct | 95% CI | Pct | 95% CI | Pct | Pct | 95% CI |
| Overall | 98.9 | (98.3,99.3) | 1.0 | (0.6,1.6) | 0.0 | (0.0,0.2) | 100.0 | 1.1 | (0.7,1.7) |
| Gender | | | | | | | |  |  |
| Doe | 99.0 | (98.3,99.4) | 1.0 | (0.6,1.6) | 0.0 | (0.0,0.2) | 100.0 | 1.0 | (0.6,1.7) |
| Buck | 98.7 | (96.3,99.5) | 1.3 | (0.5,3.7) | 0.0 | (–) | 100.0 | 1.3 | (0.5,3.7) |
| Region | | | | | | | |  |  |
| West | 99.5 | (98.9,99.7) | 0.5 | (0.3,1.1) | 0.0 | (–) | 100.0 | 0.5 | (0.3,1.1) |
| East | 98.5 | (97.4,99.2) | 1.4 | (0.8,2.5) | 0.1 | (0.0,0.4) | 100.0 | 1.5 | (0.8,2.6) |
| Primary production | | | | | | | |  |  |
| Meat | 98.7 | (97.7,99.3) | 1.3 | (0.7,2.3) | 0.0 | (–) | 100.0 | 1.3 | (0.7,2.3) |
| Dairy | 99.3 | (98.5,99.7) | 0.7 | (0.3,1.5) | 0.0 | (–) | 100.0 | 0.7 | (0.3,1.5) |
| Other | 99.1 | (97.1,99.8) | 0.7 | (0.2,2.3) | 0.1 | (0.0,0.9) | 100.0 | 0.9 | (0.2,2.9) |
| Breed | | | | | | | |  |  |
| Alpine | 99.9 | (99.5,100.0) | 0.1 | (0.0,0.5) | 0.0 | (–) | 100.0 | 0.1 | (0.0,0.5) |
| Angora | 100.0 | (–) | 0.0 | (–) | 0.0 | (–) | 100.0 | 0.0 | (–) |
| Boer | 99.5 | (98.7,99.8) | 0.5 | (0.2,1.3) | 0.0 | (–) | 100.0 | 0.5 | (0.2,1.3) |
| Cashmere | 100.0 | (–) | 0.0 | (–) | 0.0 | (–) | 100.0 | 0.0 | (–) |
| Fainting goats | 80.3 | (54.1,93.4) | 16.6 | (6.3,37.1) | 3.1 | (0.6,15.7) | 100.0 | 19.7 | (6.6,45.9) |
| Kiko | 98.7 | (96.1,99.6) | 1.3 | (0.4,3.9) | 0.0 | (–) | 100.0 | 1.3 | (0.4,3.9) |
| LaMancha | 100.0 | (–) | 0.0 | (–) | 0.0 | (–) | 100.0 | 0.0 | (–) |
| Nigerian dwarf | 99.9 | (99.7,100.0) | 0.1 | (0,0.3) | 0.0 | (–) | 100.0 | 0.1 | (0.0,0.3) |
| Nubian | 99.8 | (98.7,100.0) | 0.2 | (0.0,1.3) | 0.0 | (–) | 100.0 | 0.2 | (0.0,1.3) |
| Oberhasli | 100.0 | (–) | 0.0 | (–) | 0.0 | (–) | 100.0 | 0.0 | (–) |
| Pygmy | 100.0 | (–) | 0.0 | (–) | 0.0 | (–) | 100.0 | 0.0 | (–) |
| Pygora | 100.0 | (–) | 0.0 | (–) | 0.0 | (–) | 100.0 | 0.0 | (–) |
| Saanen | 97.4 | (93.2,99.0) | 2.6 | (1.0,6.8) | 0.0 | (–) | 100.0 | 2.6 | (1.0,6.8) |
| Sable | 100.0 | (–) | 0.0 | (–) | 0.0 | (–) | 100.0 | 0.0 | (–) |
| Savannah | 99.6 | (98.1,99.9) | 0.4 | (0.1,1.9) | 0.0 | (–) | 100.0 | 0.4 | (0.1,1.9) |
| Spanish | 99.2 | (94.6,99.9) | 0.8 | (0.1,5.4) | 0.0 | (–) | 100.0 | 0.8 | (0.1,5.4) |
| Toggenburg | 100.0 | (–) | 0.0 | (–) | 0.0 | (–) | 100.0 | 0.0 | (–) |
| Crossbred | 98.0 | (96.4,98.9) | 2.0 | (1.1,3.6) | 0.0 | (–) | 100.0 | 2.0 | (1.1,3.6) |
| Other | 97.7 | (90.2,99.5) | 2.3 | (0.5,9.8) | 0.0 | (–) | 100.0 | 2.3 | (0.5,9.8) |

**Table S1.f.** Percentage of goats by genotype at codon 211, by gender, region, primary production, and breed.

|  | Percent goats | | | | | | | | |
| --- | --- | --- | --- | --- | --- | --- | --- | --- | --- |
|  | Genotype | | | | | | | | |
|  | RR | | RQ | | QQ | | Total | RQ or QQ | |
| Breakout variable | Pct | 95% CI | Pct | 95% CI | Pct | 95% CI | Pct | Pct | 95% CI |
| Overall | 94.6 | (92.3,96.2) | 5.0 | (3.5,7.2) | 0.4 | (0.2,1.1) | 100.0 | 5.4 | (3.8,7.7) |
| Gender | | | | | | | |  |  |
| Doe | 94.4 | (92.0,96.1) | 5.4 | (3.7,7.7) | 0.2 | (0.1,0.6) | 100.0 | 5.6 | (3.9,8.0) |
| Buck | 96.4 | (89.9,98.8) | 1.5 | (0.8,2.9) | 2.1 | (0.3,11.8) | 100.0 | 3.6 | (1.2,10.1) |
| Region | | | | | | | |  |  |
| West | 97.8 | (95.5,99.0) | 1.7 | (0.9,3.0) | 0.5 | (0.1,2.3) | 100.0 | 2.2 | (1.0,4.5) |
| East | 91.9 | (88.3,94.5) | 7.7 | (5.3,11.3) | 0.3 | (0.1,1.0) | 100.0 | 8.1 | (5.5,11.7) |
| Primary production | | | | | | | |  |  |
| Meat | 95.0 | (91.7,97.0) | 4.5 | (2.7,7.5) | 0.5 | (0.1,1.9) | 100.0 | 5.0 | (3.0,8.3) |
| Dairy | 92.7 | (90.2,94.6) | 6.7 | (4.9,9.1) | 0.6 | (0.2,1.6) | 100.0 | 7.3 | (5.4,9.8) |
| Other | 95.1 | (87.4,98.2) | 4.9 | (1.8,12.6) | 0.1 | (0.0,0.3) | 100.0 | 4.9 | (1.8,12.6) |
| Breed | | | | | | | |  |  |
| Alpine | 89.0 | (83.1,93.0) | 9.7 | (6.2,14.7) | 1.3 | (0.4,4.4) | 100.0 | 11.0 | (7.0,16.9) |
| Angora | 100.0 | (–) | 0.0 | (–) | 0.0 | (–) | 100.0 | 0.0 | (–) |
| Boer | 97.9 | (95.3,99.1) | 1.6 | (0.7,3.4) | 0.5 | (0.1,1.7) | 100.0 | 2.1 | (0.9,4.7) |
| Cashmere | 98.0 | (90.5,99.6) | 2.0 | (0.4,9.5) | 0.0 | (–) | 100.0 | 2.0 | (0.4,9.5) |
| Fainting goats | 99.6 | (97.7,99.9) | 0.4 | (0.1,2.3) | 0.0 | (–) | 100.0 | 0.4 | (0.1,2.3) |
| Kiko | 85.4 | (70.5,93.5) | 14.5 | (6.4,29.4) | 0.1 | (0.0,0.6) | 100.0 | 14.6 | (6.5,29.5) |
| LaMancha | 90.8 | (81.7,95.7) | 9.1 | (4.3,18.3) | 0.0 | (0.0,0.2) | 100.0 | 9.2 | (4.3,18.3) |
| Nigerian dwarf | 99.2 | (98.0,99.6) | 0.7 | (0.3,1.9) | 0.1 | (0.0,0.5) | 100.0 | 0.8 | (0.4,2.0) |
| Nubian | 99.7 | (98.1,100.0) | 0.3 | (0.0,1.9) | 0.0 | (–) | 100.0 | 0.3 | (0.0,1.9) |
| Oberhasli | 71.5 | (38.5,90.9) | 28.5 | (9.0,61.4) | 0.1 | (0.0,0.4) | 100.0 | 28.5 | (9.1,61.5) |
| Pygmy | 100.0 | (–) | 0.0 | (–) | 0.0 | (–) | 100.0 | 0.0 | (–) |
| Pygora | 100.0 | (–) | 0.0 | (–) | 0.0 | (–) | 100.0 | 0.0 | (–) |
| Saanen | 91.9 | (84.6,95.9) | 7.8 | (3.9,15.1) | 0.2 | (0.1,0.9) | 100.0 | 8.1 | (4.1,15.4) |
| Sable | 40.3 | (9.1,82.0) | 58.3 | (16.6,90.7) | 1.5 | (0.2,10.8) | 100.0 | 59.7 | (18.0,90.9) |
| Savannah | 100.0 | (–) | 0.0 | (–) | 0.0 | (–) | 100.0 | 0.0 | (–) |
| Spanish | 92.6 | (84.4,96.7) | 7.4 | (3.3,15.6) | 0.0 | (–) | 100.0 | 7.4 | (3.3,15.6) |
| Toggenburg | 99.5 | (96.4,99.9) | 0.5 | (0.1,3.6) | 0.0 | (–) | 100.0 | 0.5 | (0.1,3.6) |
| Crossbred | 91.6 | (84.0,95.7) | 7.5 | (3.7,14.5) | 0.9 | (0.2,5.3) | 100.0 | 8.4 | (4.3,16.0) |
| Other | 85.9 | (55.6,96.7) | 13.3 | (2.8,44.8) | 0.8 | (0.2,3.8) | 100.0 | 14.1 | (3.3,44.4) |

**Table S1.g.** Percentage of goats by genotype at codon 222, by gender, region, primary production, and breed.

|  | Percent goats | | | | | | | | |
| --- | --- | --- | --- | --- | --- | --- | --- | --- | --- |
|  | Genotype | | | | | | | | |
|  | QQ | | QK | | KK | | Total | QK or KK | |
| Breakout variable | Pct | 95% CI | Pct | 95% CI | Pct | 95% CI | Pct | Pct | 95% CI |
| Overall | 99.4 | (98.8,99.7) | 0.6 | (0.3,1.2) | 0.0 | (–) | 100.0 | 0.6 | (0.3,1.2) |
| Gender | | | | | | | |  |  |
| Doe | 99.4 | (98.7,99.7) | 0.6 | (0.3,1.3) | 0.0 | (–) | 100.0 | 0.6 | (0.3,1.3) |
| Buck | 99.0 | (97.3,99.7) | 1.0 | (0.3,2.7) | 0.0 | (–) | 100.0 | 1.0 | (0.3,2.7) |
| Region | | | | | | | |  |  |
| West | 99.9 | (99.6,100.0) | 0.1 | (0.0,0.4) | 0.0 | (–) | 100.0 | 0.1 | (0.0,0.4) |
| East | 99.0 | (97.9,99.5) | 1.0 | (0.5,2.1) | 0.0 | (–) | 100.0 | 1.0 | (0.5,2.1) |
| Primary production | | | | | | | |  |  |
| Meat | 99.4 | (98.2,99.8) | 0.6 | (0.2,1.8) | 0.0 | (–) | 100.0 | 0.6 | (0.2,1.8) |
| Dairy | 98.9 | (97.5,99.5) | 1.1 | (0.5,2.5) | 0.0 | (–) | 100.0 | 1.1 | (0.5,2.5) |
| Other | 99.9 | (99.0, 100.0) | 0.1 | (0.0,1.0) | 0.0 | (–) | 100.0 | 0.1 | (0.0,1.0) |
| Breed | | | | | | | |  |  |
| Alpine | 99.6 | (98.7,99.9) | 0.4 | (0.1,1.3) | 0.0 | (–) | 100.0 | 0.4 | (0.1,1.3) |
| Angora | 100.0 | (–) | 0.0 | (–) | 0.0 | (–) | 100.0 | 0.0 | (–) |
| Boer | 99.9 | (99.6,100.0) | 0.1 | (0.0,0.4) | 0.0 | (–) | 100.0 | 0.1 | (0.0,0.4) |
| Cashmere | 100.0 | (–) | 0.0 | (–) | 0.0 | (–) | 100.0 | 0.0 | (–) |
| Fainting goats | 100.0 | (–) | 0.0 | (–) | 0.0 | (–) | 100.0 | 0.0 | (–) |
| Kiko | 100.0 | (–) | 0.0 | (–) | 0.0 | (–) | 100.0 | 0.0 | (–) |
| LaMancha | 99.9 | (99.4,100.0) | 0.1 | (0.0,0.6) | 0.0 | (–) | 100.0 | 0.1 | (0.0,0.6) |
| Nigerian dwarf | 100.0 | (99.7,100.0) | 0.0 | (0.0,0.3) | 0.0 | (–) | 100.0 | 0.0 | (0.0,0.3) |
| Nubian | 100.0 | (–) | 0.0 | (–) | 0.0 | (–) | 100.0 | 0.0 | (–) |
| Oberhasli | 76.9 | (31.3,96.1) | 23.1 | (3.9,68.7) | 0.0 | (–) | 100.0 | 23.1 | (3.9,68.7) |
| Pygmy | 100.0 | (–) | 0.0 | (–) | 0.0 | (–) | 100.0 | 0.0 | (–) |
| Pygora | 100.0 | (–) | 0.0 | (–) | 0.0 | (–) | 100.0 | 0.0 | (–) |
| Saanen | 99.9 | (99.7,100.0) | 0.1 | (0.0,0.3) | 0.0 | (–) | 100.0 | 0.1 | (0.0,0.3) |
| Sable | 100.0 | (–) | 0.0 | (–) | 0.0 | (–) | 100.0 | 0.0 | (–) |
| Savannah | 99.9 | (99.5,100.0) | 0.1 | (0.0,0.5) | 0.0 | (–) | 100.0 | 0.1 | (0.0,0.5) |
| Spanish | 99.2 | (94.5,99.9) | 0.8 | (0.1,5.5) | 0.0 | (–) | 100.0 | 0.8 | (0.1,5.5) |
| Toggenburg | 78.0 | (57.5,90.3) | 22.0 | (9.7,42.5) | 0.0 | (–) | 100.0 | 22.0 | (9.7,42.5) |
| Crossbred | 99.3 | (98.3,99.7) | 0.7 | (0.3,1.7) | 0.0 | (–) | 100.0 | 0.7 | (0.3,1.7) |
| Other | 96.1 | (83.4,99.2) | 3.9 | (0.8,16.6) | 0.0 | (–) | 100.0 | 3.9 | (0.8,16.6) |

**Table S1.h.** Percentage of goats by genotype at codon 240, by gender, region, primary production, and breed.

|  | Percent goats | | | | | | | | |
| --- | --- | --- | --- | --- | --- | --- | --- | --- | --- |
|  | Genotype | | | | | | | | |
|  | PP | | PS | | SS | | Total | PS or SS | |
| Breakout variable | Pct | 95% CI | Pct | 95% CI | Pct | 95% CI | Pct | Pct | 95% CI |
| Overall | 54.0 | (50.6,57.3) | 36.7 | (33.6,39.8) | 9.3 | (7.9,11.0) | 100.0 | 46.0 | (42.7,49.4) |
| Gender | | | | | | | |  |  |
| Doe | 54.4 | (50.7,57.9) | 36.7 | (33.5,40.0) | 8.9 | (7.5,10.6) | 100.0 | 45.7 | (42.1,49.3) |
| Buck | 50.1 | (41.3,58.9) | 36.5 | (28.1,45.8) | 13.4 | (7.5,23.0) | 100.0 | 49.9 | (41.1,58.7) |
| Region | | | | | | | |  |  |
| West | 56.8 | (51.4,62.1) | 33.9 | (29.3,38.8) | 9.3 | (7.1,12.0) | 100.0 | 43.2 | (37.9,48.6) |
| East | 51.7 | (47.6,55.7) | 38.9 | (35.2,42.8) | 9.4 | (7.5,11.7) | 100.0 | 48.3 | (44.3,52.4) |
| Primary production | | | | | | | |  |  |
| Meat | 56.2 | (51.2,60.9) | 34.6 | (30.4,39.1) | 9.2 | (7.3,11.6) | 100.0 | 43.9 | (39.1,48.8) |
| Dairy | 50.7 | (46.4,55.0) | 40.1 | (36.7,43.7) | 9.2 | (6.8,12.2) | 100.0 | 49.3 | (45.0,53.6) |
| Other | 51.4 | (45.3,57.5) | 38.8 | (32.4,45.5) | 9.8 | (6.8,13.8) | 100.0 | 48.6 | (42.5,54.7) |
| Breed | | | | | | | |  |  |
| Alpine | 48.8 | (38.6,59.1) | 42.0 | (34.9,49.5) | 9.2 | (4.9,16.4) | 100.0 | 51.2 | (40.9,61.4) |
| Angora | 49.3 | (38.8,59.8) | 35.2 | (25.5,46.3) | 15.5 | (9.2,24.9) | 100.0 | 50.7 | (40.2,61.2) |
| Boer | 48.7 | (44.1,53.3) | 40.8 | (36.8,44.9) | 10.5 | (8.4,13.1) | 100.0 | 51.4 | (46.8,56.0) |
| Cashmere | 70.3 | (44.3,87.5) | 29.7 | (12.5,55.7) | 0.0 | (–) | 100.0 | 29.7 | (12.5,55.7) |
| Fainting goats | 61.2 | (46.2,74.4) | 30.7 | (20.8,42.7) | 8.1 | (2.9,20.6) | 100.0 | 38.8 | (25.6,53.8) |
| Kiko | 53.7 | (41.8,65.2) | 40.9 | (29.9,52.9) | 5.4 | (2.8,10.1) | 100.0 | 46.3 | (34.8,58.2) |
| LaMancha | 68.5 | (56.7,78.3) | 26.7 | (17.6,38.3) | 4.8 | (1.4,14.7) | 100.0 | 31.5 | (21.7,43.3) |
| Nigerian dwarf | 53.0 | (44.1,61.7) | 32.0 | (24.2,41.0) | 15.0 | (9.2,23.5) | 100.0 | 47.0 | (38.3,55.9) |
| Nubian | 53.4 | (42.4,64.1) | 32.5 | (25.4,40.5) | 14.1 | (7.2,25.9) | 100.0 | 46.6 | (35.9,57.6) |
| Oberhasli | 4.1 | (1.3,12.0) | 17.7 | (8.3,33.8) | 78.3 | (60.0,89.6) | 100.0 | 95.9 | (88.0,98.7) |
| Pygmy | 47.2 | (30.4,64.7) | 41.4 | (30.3,53.4) | 11.4 | (2.6,37.8) | 100.0 | 52.8 | (35.3,69.6) |
| Pygora | 5.2 | (1.2,20.2) | 60.9 | (43.4,75.9) | 34.0 | (15.7,58.7) | 100.0 | 94.8 | (79.8,98.8) |
| Saanen | 66.2 | (54.4,76.4) | 30.5 | (21.1,42.0) | 3.2 | (1.3,7.6) | 100.0 | 33.8 | (23.6,45.6) |
| Sable | 14.3 | (2.7,49.8) | 81.5 | (43.2,96.2) | 4.2 | (0.7,20.6) | 100.0 | 85.7 | (50.2,97.3) |
| Savannah | 52.8 | (27.4,76.9) | 42.9 | (22.8,65.6) | 4.3 | (0.9,17.9) | 100.0 | 47.2 | (23.1,72.6) |
| Spanish | 64.7 | (54.8,73.5) | 29.8 | (21.4,39.8) | 5.5 | (2.8,10.6) | 100.0 | 35.3 | (26.5,45.2) |
| Toggenburg | 70.6 | (52.6,83.9) | 27.4 | (14.7,45.2) | 2.0 | (0.3,12.9) | 100.0 | 29.4 | (16.1,47.4) |
| Crossbred | 51.2 | (43.6,58.8) | 40.1 | (32.7,48.0) | 8.6 | (5.7,12.8) | 100.0 | 48.8 | (41.2,56.4) |
| Other | 46.6 | (30.7,63.2) | 35.4 | (25.1,47.3) | 18.0 | (8.5,34.2) | 100.0 | 53.4 | (36.8,69.3) |

**Table S1.i.** Percentage of goats by presence of S146, D146, or K222 genotypes, by gender, region, primary production, and breed.

|  | Percent goats | | | |  |
| --- | --- | --- | --- | --- | --- |
|  | Genotype | | | |  |
|  | Present | | Absent | | Total |
| Breakout variable | Pct | 95% CI | Pct | 95% CI | Pct |
| Overall | 33.8 | (29.6,38.3) | 66.2 | (61.7,70.4) | 100.0 |
| Gender | | | | |  |
| Doe | 34.7 | (30.3,39.4) | 65.3 | (60.6,69.7) | 100.0 |
| Buck | 24.4 | (17.4,33.3) | 75.6 | (66.7,82.6) | 100.0 |
| Region | | | | |  |
| West | 37.7 | (30.4,45.6) | 62.3 | (54.4,69.6) | 100.0 |
| East | 30.7 | (26.3,35.4) | 69.3 | (64.6,73.7) | 100.0 |
| Primary production | | | | |  |
| Meat | 41.7 | (36.5,47.2) | 58.3 | (52.8,63.5) | 100.0 |
| Dairy | 18.2 | (14.2,23.0) | 81.8 | (77.0,85.8) | 100.0 |
| Other | 27.6 | (19.0,38.3) | 72.4 | (61.7,81.0) | 100.0 |
| Breed | | | | |  |
| Alpine | 4.2 | (1.8,9.6) | 95.8 | (90.4,98.2) | 100.0 |
| Angora | 0.9 | (0.1,6.5) | 99.1 | (93.5,99.9) | 100.0 |
| Boer | 47.0 | (42.9,51.1) | 53.0 | (48.9,57.1) | 100.0 |
| Cashmere | 23.0 | (5.6,59.9) | 77.0 | (40.1,94.4) | 100.0 |
| Fainting goats | 3.4 | (0.6,16.1) | 96.6 | (83.9,99.4) | 100.0 |
| Kiko | 17.2 | (8.0,33.2) | 82.8 | (66.8,92.0) | 100.0 |
| LaMancha | 35.9 | (23.1,51.1) | 64.1 | (48.9,76.9) | 100.0 |
| Nigerian dwarf | 0.7 | (0.3,2.1) | 99.3 | (97.9,99.7) | 100.0 |
| Nubian | 38.6 | (29.8,48.2) | 61.4 | (51.8,70.2) | 100.0 |
| Oberhasli | 24.8 | (4.8,68.2) | 75.2 | (31.8,95.2) | 100.0 |
| Pygmy | 0.6 | (0.1,3.4) | 99.4 | (96.6,99.9) | 100.0 |
| Pygora | 1.1 | (0.1,10.1) | 98.9 | (89.9,99.9) | 100.0 |
| Saanen | 2.9 | (1.1,7.5) | 97.1 | (92.5,98.9) | 100.0 |
| Sable | 8.5 | (0.9,47.7) | 91.5 | (52.3,99.1) | 100.0 |
| Savannah | 65.5 | (30.8,89.0) | 34.5 | (11.0,69.2) | 100.0 |
| Spanish | 43.4 | (30.9,56.7) | 56.6 | (43.3,69.1) | 100.0 |
| Toggenburg | 35.0 | (11.3,69.4) | 65.0 | (30.6,88.7) | 100.0 |
| Crossbred | 38.3 | (32.2,44.7) | 61.7 | (55.3,67.8) | 100.0 |
| Other | 16.2 | (8.8,27.9) | 83.8 | (72.1,91.2) | 100.0 |
