# Supplementary material for: Large-scale survey of prion protein genetic variability in scrapie disease-free goats from the United States
Source: PLoS One. 2021 Jul 19;16(7):e0254998. doi: 10.1371/journal.pone.0254998 (PMC8289333; doi:10.1371/journal.pone.0254998)
Supplement: S2 Table — a. Percentage of operations by genotype at codon 127, by region and primary production. b. Percentage of operations by genotype at codon 127, by region and primary production. c. Percentage of operations by genotype at codon 143, by region and primary production. d. Percentage of operations by genotype at codon 146, by region and primary production. e. Percentage of operations by genotype at codon 154, by region and primary production. f. Percentage of operations by genotype at codon 211, by region and primary production. g. Percentage of operations by genotype at codon 222, by region and primary production. h. Percentage of operations by genotype at codon 240, by region and primary production. i. Percentage of operations by presence of S146, D146, or K222 genotypes, by gender, region, primary production, and breed. (DOCX) [file pone.0254998.s002.docx]

# Percentage of operations by codon, by genotype, and by breakout variables

**Table S2.a.** Percentage of operations by genotype at codon 127, by region and primary production.

|  | Percent operations | | | | | | | |
| --- | --- | --- | --- | --- | --- | --- | --- | --- |
|  | Genotype | | | | | | | |
|  | GG | | GS | | SS | | GS or SS | |
| Breakout variable | Pct | 95% CI | Pct | 95% CI | Pct | 95% CI | Pct | 95% CI |
| Overall | 100.0 | (–) | 2.3 | (1.0, 5.2) | 0.0 | (0.0, 0.1) | 2.4 | (1.0,5.2) |
| Region | | | | | | | | |
| West | 100.0 | (–) | 0.6 | (0.1,2.2) | 0.0 | (–) | 0.6 | (0.1,2.2) |
| East | 100.0 | (–) | 3.1 | (1.3,7.3) | 0.0 | (0.0,0.2) | 3.1 | (1.3,7.3) |
| Primary production | | | | | | | | |
| Meat | 100.0 | (–) | 0.2 | (0.0,1.6) | 0.0 | (–) | 0.2 | (0.0,1.6) |
| Dairy | 100.0 | (–) | 7.1 | (2.5,18.8) | 0.1 | (0.0,0.5) | 7.2 | (2.6,18.8) |
| Other | 100.0 | (–) | 2.3 | (0.7,7.7) | 0.0 | (–) | 2.3 | (0.7,7.7) |

**Table S2.b.** Percentage of operations by genotype at codon142, by region and primary production.

|  | Percent operations | | | | | | | |
| --- | --- | --- | --- | --- | --- | --- | --- | --- |
|  | Genotype | | | | | | | |
|  | II | | IM | | MM | | IM or MM | |
| Breakout variable | Pct | 95% CI | Pct | 95% CI | Pct | 95% CI | Pct | 95% CI |
| Overall | 99.6 | (98.1,99.9) | 26.8 | (21.1,33.4) | 3.9 | (2.0,7.5) | 28.0 | (22.2,34.5) |
| Region | | | | | | | | |
| West | 100.0 | (–) | 23.2 | (14.6,35.0) | 1.2 | (0.6,2.4) | 23.6 | (14.9,35.3) |
| East | 99.4 | (97.4,99.9) | 28.4 | (21.3,36.6) | 5.0 | (2.4,10.4) | 29.8 | (22.8,38.0) |
| Primary production | | | | | | | | |
| Meat | 100.0 | (–) | 22.9 | (15.4,32.7) | 2.2 | (0.5,9.4) | 23.5 | (15.9,33.3) |
| Dairy | 99.4 | (97.1,99.9) | 38.9 | (27.5,51.5) | 9.9 | (4.1,22.1) | 42.6 | (31.4,54.6) |
| Other | 98.8 | (92.0,99.8) | 24.0 | (15.0,36.1) | 1.8 | (0.5,6.3) | 24.0 | (15.0,36.1) |

**Table S2.c.** Percentage of operations by genotype at codon 143, by region and primary production.

|  | Percent operations | | | | | | | |
| --- | --- | --- | --- | --- | --- | --- | --- | --- |
|  | Genotype | | | | | | | |
|  | HH | | HR | | RR | | HR or RR | |
| Breakout variable | Pct | 95% CI | Pct | 95% CI | Pct | 95% CI | Pct | 95% CI |
| Overall | 99.8 | (99.1, 99.9) | 48.7 | (42.0, 55.4) | 8.1 | (5.3, 12.2) | 49.8 | (42.9,56.6) |
| Region | | | | | | | | |
| West | 100.0 | (–) | 48.4 | (36.9,60.1) | 7.9 | (3.9,15.2) | 52.0 | (39.4,64.3) |
| East | 99.7 | (98.6,99.9) | 48.8 | (40.7,56.9) | 8.2 | (4.8,13.6) | 48.8 | (40.7,57.0) |
| Primary production | | | | | | | | |
| Meat | 100.0 | (–) | 58.0 | (47.0,68.2) | 9.9 | (5.8,16.3) | 59.9 | (48.9,70.0) |
| Dairy | 98.9 | (95.8,99.7) | 34.4 | (23.1,47.9) | 3.0 | (1.3,6.8) | 34.4 | (23.1,47.9) |
| Other | 100.0 | (–) | 42.5 | (30.5,55.5) | 9.1 | (4.0,19.2) | 42.9 | (30.9,55.9) |

**Table S2.d.** Percentage of operations by genotype at codon 146, by region and primary production.

|  | Percent operations | | | | | | | | | | | | | |
| --- | --- | --- | --- | --- | --- | --- | --- | --- | --- | --- | --- | --- | --- | --- |
|  | Genotype | | | | | | | | | | | | | |
|  | NN | | NS | | ND | | SS | | DD | | SD | | Any non-NN | |
| Breakout variable | Pct | 95% CI | Pct | 95% CI | Pct | 95% CI | Pct | 95% CI | Pct | 95% CI | Pct | 95% CI | Pct | 95% CI |
| Overall | 94.1 | (88.7,97.0) | 68.6 | (62.5,74.1) | 4.3 | (2.4,7.3) | 19.6 | (14.5,26.0) | 1.2 | (0.4,3.8) | 1.0 | (0.3,3.3) | 72.3 | (66.3,77.5) |
| Region | | | | | | | | | | | | | | |
| West | 91.5 | (78.2,97.0) | 70.1 | (58.8,79.4) | 7.7 | (3.7,15.3) | 25.6 | (16.3,37.9) | 2.7 | (0.6,11.1) | 2.6 | (0.6,11.1) | 75.6 | (64.9,83.8) |
| East | 95.2 | (88.6,98.1) | 67.9 | (60.5,74.5) | 2.7 | (1.1,6.6) | 17.0 | (11.2,25.0) | 0.6 | (0.1,3.4) | 0.3 | (0.1,1.0) | 70.8 | (63.5,77.2) |
| Primary production | | | | | | | | | | | | | | |
| Meat | 94.2 | (85.3,97.8) | 84.2 | (75.7,90.2) | 6.1 | (3.2,11.4) | 26.3 | (18.3,36.1) | 2.2 | (0.6,7.3) | 1.6 | (0.4,6.5) | 88.9 | (80.7,93.9) |
| Dairy | 95.7 | (81.3,99.1) | 49.7 | (37.9,61.6) | 0.6 | (0.1,3.4) | 8.7 | (3.6,19.3) | 0.0 | (–) | 0.5 | (0.1,3.6) | 51.0 | (39.1,62.7) |
| Other | 92.6 | (78.5,97.7) | 53.8 | (40.1,66.9) | 3.8 | (1.0,13.0) | 15.9 | (7.9,29.5) | 0.5 | (0.1,3.6) | 0.1 | (0.0,0.3) | 57.6 | (43.7,70.4) |

**Table S2.e.** Percentage of operations by genotype at codon 154, by region and primary production.

|  | Percent operations | | | | | | | |
| --- | --- | --- | --- | --- | --- | --- | --- | --- |
|  | Genotype | | | | | | | |
|  | RR | | RH | | HH | | RH or HH | |
| Breakout variable | Pct | 95% CI | Pct | 95% CI | Pct | 95% CI | Pct | 95% CI |
| Overall | 100.0 | (–) | 5.6 | (3.3,9.3) | 0.3 | (0.0,2.0) | 5.6 | (3.3,9.3) |
| Region | | | | | | | | |
| West | 100.0 | (–) | 2.7 | (1.3,5.3) | 0.0 | (–) | 2.7 | (1.3,5.3) |
| East | 100.0 | (–) | 6.8 | (3.7,12.2) | 0.4 | (0.1,2.8) | 6.8 | (3.7,12.2) |
| Primary production | | | | | | | | |
| Meat | 100.0 | (–) | 6.0 | (3.0,11.7) | 0.0 | (–) | 6.0 | (3.0,11.7) |
| Dairy | 100.0 | (–) | 3.5 | (1.4,8.5) | 0.0 | (–) | 3.5 | (1.4,8.5) |
| Other | 100.0 | (–) | 6.6 | (2.2,17.8) | 1.1 | (0.2,7.3) | 6.6 | (2.2,17.8) |

**Table S2.f.** Percentage of operations by genotype at codon 211, by region and primary production.

|  | Percent operations | | | | | | | |
| --- | --- | --- | --- | --- | --- | --- | --- | --- |
|  | Genotype | | | | | | | |
|  | RR | | RQ | | QQ | | RQ or QQ | |
| Breakout variable | Pct | 95% CI | Pct | 95% CI | Pct | 95% CI | Pct | 95% CI |
| Overall | 100.0 | (99.9,100.0) | 15.0 | (11.3,19.7) | 3.4 | (1.4,7.9) | 15.1 | (11.4,19.8) |
| Region | | | | | | | | |
| West | 100.0 | (99.8,100.0) | 8.4 | (4.9,14.1) | 2.8 | (0.7,11.3) | 8.6 | (5.0,14.3) |
| East | 100.0 | (99.8,100.0) | 17.9 | (12.8,24.4) | 3.6 | (1.2,10.1) | 17.9 | (12.8,24.4) |
| Primary production | | | | | | | | |
| Meat | 100.0 | (–) | 12.5 | (7.8,19.5) | 1.9 | (0.3,9.6) | 12.6 | (7.8,19.6) |
| Dairy | 99.9 | (99.7,100.0) | 23.5 | (16.3,32.7) | 7.4 | (2.1,22.8) | 23.7 | (16.5,32.9) |
| Other | 99.9 | (99.4,100.0) | 12.6 | (6.3,23.4) | 2.8 | (0.5,14.1) | 12.6 | (6.3,23.4) |

**Table S2.g.** Percentage of operations by genotype at codon 222, by region and primary production.

|  | Percent operations | | | | | | | |
| --- | --- | --- | --- | --- | --- | --- | --- | --- |
|  | Genotype | | | | | | | |
|  | QQ | | QK | | KK | | QK or KK | |
| Breakout variable | Pct | 95% CI | Pct | 95% CI | Pct | 95% CI | Pct | 95% CI |
| Overall | 100.0 | (–) | 2.1 | (1.0,4.6) | 0.0 | (–) | 2.1 | (1.0,4.6) |
| Region | | | | | | | | |
| West | 100.0 | (–) | 3.0 | (0.8,10.6) | 0.0 | (–) | 3.0 | (0.8,10.6) |
| East | 100.0 | (–) | 1.8 | (0.7,4.4) | 0.0 | (–) | 1.8 | (0.7,4.4) |
| Primary production | | | | | | | | |
| Meat | 100.0 | (–) | 3.1 | (1.1,8.3) | 0.0 | (–) | 3.1 | (1.1,8.3) |
| Dairy | 100.0 | (–) | 1.9 | (1.1,3.2) | 0.0 | (–) | 1.9 | (1.1,3.2) |
| Other | 100.0 | (–) | 0.4 | (0.1,2.3) | 0.0 | (–) | 0.4 | (0.1,2.3) |

**Table S.2.h.** Percentage of operations by genotype at codon 240, by region and primary production.

|  | Percent operations | | | | | | | |
| --- | --- | --- | --- | --- | --- | --- | --- | --- |
|  | Genotype | | | | | | | |
|  | PP | | PS | | SS | | PS or SS | |
| Breakout variable | Pct | 95% CI | Pct | 95% CI | Pct | 95% CI | Pct | 95% CI |
| Overall | 95.0 | (90.5,97.5) | 90.1 | (84.5,93.8) | 47.0 | (40.4,53.7) | 93.3 | (88.5,96.2) |
| Region | | | | | | | | |
| West | 93.2 | (81.2,97.7) | 80.8 | (66.6,89.8) | 52.4 | (39.5,65.0) | 87.8 | (75.1,94.5) |
| East | 95.8 | (90.5,98.2) | 94.1 | (88.3,97.1) | 44.6 | (37.1,52.4) | 95.7 | (90.8,98.0) |
| Primary production | | | | | | | | |
| Meat | 97.6 | (90.6,99.4) | 89.2 | (79.7,94.5) | 47.3 | (37.9,56.9) | 93.7 | (85.5,97.4) |
| Dairy | 96.4 | (91.6,98.5) | 90.1 | (78.3,95.8) | 38.8 | (26.4,52.8) | 92.5 | (81.7,97.1) |
| Other | 88.6 | (74.0,95.5) | 91.7 | (82.1,96.4) | 53.5 | (40.0,66.4) | 93.3 | (83.2,97.5) |

**Table S2.i.** Percentage of operations by presence of S146, D146, or K222 genotypes, by gender, region, primary production, and breed.

|  | Percent operations | | | |
| --- | --- | --- | --- | --- |
|  | Genotype | | | |
|  | Present | | Absent | |
| Breakout variable | Pct | 95% CI | Pct | 95% CI |
| Overall | 72.8 | (66.9,78.0) | 27.2 | (22.0,33.1) |
| Region | | | | |
| West | 76.0 | (65.2,84.2) | 24.0 | (15.8,34.8) |
| East | 71.5 | (64.2,77.8) | 28.5 | (22.2,35.8) |
| Primary production | | | | |
| Meat | 89.5 | (81.3,94.4) | 10.5 | (5.6,18.7) |
| Dairy | 52.1 | (40.2,63.7) | 47.9 | (36.3,59.8) |
| Other | 57.6 | (43.7,70.4) | 42.4 | (29.6,56.3) |
